# Mitochondrial damage and activation of the cytosolic DNA sensor cGAS–STING pathway lead to cardiac pyroptosis and hypertrophy in diabetic cardiomyopathy mice

**DOI:** 10.1038/s41420-022-01046-w

**Published:** 2022-05-11

**Authors:** Meiling Yan, Yun Li, Qingmao Luo, Wenru Zeng, Xiaoqi Shao, Lun Li, Qing Wang, Dongwei Wang, Yue Zhang, Hongtao Diao, Xianglu Rong, Yunlong Bai, Jiao Guo

**Affiliations:** 1 The Center for Drug Research and Development, Guangdong Pharmaceutical University, Guangzhou, 510006 China; 2 Guangdong Metabolic Disease Research Center of Integrated Chinese and Western Medicine, Guangzhou, 510006 China; 3 Key Laboratory of Glucolipid Metabolic Disorder, Ministry of Education of China, Guangzhou, 510006 China; 4 Institute of Chinese Medicine, Guangdong Pharmaceutical University, Guangzhou, 510006 China; 5 Guangdong TCM Key Laboratory for Metabolic Diseases, Guangzhou, 510006 China; 6 Department of Pharmacology (State-Province Key Laboratories of Biomedicine-Pharmaceutics of China, Key Laboratory of Cardiovascular Medicine Research, Ministry of Education), College of Pharmacy, Harbin Medical University, Harbin, China; 7 Translational Medicine Research and Cooperation Center of Northern China, Chronic Disease Research Institute, Heilongjiang Academy of Medical Sciences, Harbin, China

**Keywords:** Cardiac hypertrophy, Type 2 diabetes

## Abstract

Diabetic cardiomyopathy (DCM) is a serious cardiac complication of diabetes that currently lacks specific treatment. The cyclic GMP-AMP synthase (cGAS)-stimulator of interferon genes (STING) signaling pathway has been suggested to contribute to the pathogenesis of cardiovascular diseases. However, whether cGAS-STING is involved in the development of DCM has not been established. Our study aimed to determine the role of cGAS-STING in the initiation of nucleotide-binding oligomerization domain-like receptor pyrin domain containing 3 (NLRP3) inflammasome-induced cardiac pyroptosis and chronic inflammation during the pathogenesis of DCM. C57BL/6J mice were preinjected with adeno-associated virus 9 (AAV9) intravenously via the tail vein to specifically knock down myocardial STING. After four weeks, mice with myocardium-specific knockdown of STING received injections of streptozotocin (STZ; 50 mg/kg) and a high-fat diet to induce diabetes. Measurements included echocardiography, immunohistochemical analyses, wheat germ agglutinin (WGA) staining, and western blotting. Here, we showed that the cGAS-STING signaling pathway was activated in diabetic hearts, which was indicated by the increased phosphorylation of TANK-binding kinase 1 (TBK1) and interferon (IFN) regulatory factor 3 (IRF3), leading to the activation of the NLRP3 inflammasome in the hearts of diabetic mice and proinflammatory cytokine release into serum. Moreover, STING knockdown via adeno-associated virus-9 (AAV9) in diabetic mouse heart alleviated cardiac pyroptosis and the inflammatory response, prevented diabetes-induced hypertrophy, and restored cardiac function. Mechanistically, we showed that palmitic acid (PA)-induced lipotoxicity impairs mitochondrial homeostasis, producing excessive mitochondrial reactive oxygen species (mtROS), which results in oxidative damage to mitochondrial DNA (mtDNA) and its release into the cytoplasm while switching on cGAS-STING-mediated pyroptosis in cardiomyocytes, thereby worsening the progression of diabetic cardiomyopathy. Our study demonstrated that activation of the cGAS-STING pathway caused by mitochondrial oxidative damage and mtDNA escape induced by free fatty acids promoted pyroptosis and proinflammatory responses in cardiomyocytes in a NLRP3 inflammasome-dependent manner, thus promoting myocardial hypertrophy during the progression of DCM.

## Background

Diabetic cardiomyopathy (DCM) is one of the vital causes of mortality in diabetic patients and is distinguished by cardiac hypertrophy and fibrosis that is independent of vascular complications during diabetes [1]. The multiple molecular mechanisms of DCM remain unclear, leading to the absence of patient-specific treatment guidance [2]. Current treatment strategies for DCM are limited to lifestyle interventions and glycemic management. However, intensive glycemic control is not enough to reduce the risk of heart failure in diabetic patients, suggesting that diabetes-related cardiac insufficiency may be caused by other metabolic triggers [3].

Physiologically, intracellular adenosine triphosphate (ATP) production is dependent on mitochondrial oxidative phosphorylation, 30% through glycolysis and 70% through fatty acid oxidation in cardiomyocytes [4]. However, an excess of free fatty acids (FFAs) and aberrant β-oxidation in the diabetic heart lead to increased lipid metabolites, which mediate cardiac lipotoxicity and have many detrimental consequences [5–7]. During the phase of diabetes, increased FFAs oxidation reduces cardiac efficiency and contributes to ventricular dysfunction by increasing the generation of reactive oxygen species (ROS) and toxic lipid intermediates, which in turn impairs multiple cellular components, such as mitochondria and DNA, by the oxidative transformation of lipids into lipid peroxides. Hence, the accumulated lipid metabolites in cardiomyocytes (CMs) trigger programmed cell death and consequently drive the development of heart failure [8, 9]. Therefore, inhibition of FFAs accumulation and oxidation are considered potential therapeutic strategies for DCM treatment [10].

Pyroptosis, as a typical kind of cell death, is closely related to the activation of inflammation [11]. It has been reported that the nucleotide-binding oligomerization domain-like receptor pyrin domain containing 3 (NLRP3) inflammasome can be activated by lipid toxicity, thereby promoting the transformation of pro-caspase-1 into caspase-1. This converts pro-interleukin-1β (pro-IL-1β) and pro-interleukin-18 (pro-IL-18) into mature IL-1β/IL-18 and simultaneously cleaves gasdermin D (GSDMD) into cleaved *N*-terminal of GSDMD (GSDMD-N), which is essential during the process of pyroptosis as it promotes the secretion of mature IL-1β/IL-18 and destroys the plasma membrane [12–14]. More importantly, it has been proven that activation of the NLRP3 inflammasome mediating the release of proinflammatory cytokines into CMs can promote the deposition of collagen and the formation of left ventricular (LV) hypertrophy, fibrosis, and diastolic dysfunction with normal ejection fraction, which further develops into heart failure with preserved ejection fraction (HFpEF), whereas others eventually develop into heart failure with reduced ejection fraction (HFrEF) [15, 16]. Therefore, it is essential to explore the deeper mechanisms by which myocardial pyroptosis is initiated during DCM.

The cGAS-STING signaling pathway, comprising the cyclic GMP-AMP synthase (cGAS) and the cyclic GMP-AMP receptor stimulator of interferon genes (STING), is an evolutionarily conserved defense mechanism that detects pathogenic DNA [17], triggering an innate immune reaction involving a strong type I interferon response [18]. Briefly, cGAS functions as a double-stranded DNA (dsDNA) sensor that surveys cytosolic DNA and then produces cGAMP, which binds to STING and activates the transcription factor IRF3 and nuclear factor-κ-gene binding (NF-κB) through the kinases TBK1 and I-kappa-B kinase (IKK), respectively [19, 20]. In addition to playing a critical role in the antiviral immune response, cGAS has also been shown to be involved in some other important biological processes, such as macular degeneration [21], cellular senescence [22, 23], myocardial infarction-related inflammation [24], and macrophage transformation [25]. Notably, in addition to sensing microbial DNA, cGAS has been proven to sense endogenous DNA, including extranuclear chromatin resulting from genotoxic stress and DNA released from mitochondria [26]. Recently, the interplay between the cGAS-STING axis and NLRP3 has been revealed [27]. In sepsis-induced cardiomyopathy, STING activation in cardiomyocytes treated with LPS triggered IRF3-dependent NLRP3 activation, promoting cardiac injury. Moreover, STING-dependent NLRP3 activation in macrophages mediates innate immune activation in aged mouse livers during ischemia and reperfusion [28]. However, whether the cGAS-STING signaling pathway initiates NLRP3 inflammasome activation to aggravate the pathological progression of DCM remains unclear.

In the present study, we have proven that the cGAS-STING signaling pathway was activated by mitochondrial oxidative damage induced cytoplasmic mitochondrial DNA (mtDNA) in the hearts of DCM mice, which mediated cardiac injury. Mechanistically, our results demonstrated that lipotoxicity induced by FFAs triggers pyroptosis of myocardial cells through the activation of NLRP3 inflammasome in a cGAS-STING-dependent manner, which contributes to the sterile inflammatory response and myocardial hypertrophy.

## Results

### Escape of mitochondrial DNA (mtDNA) into the cytosol and cGAS-STING pathway activation in the hearts of DCM mice

**Fig. 1:**
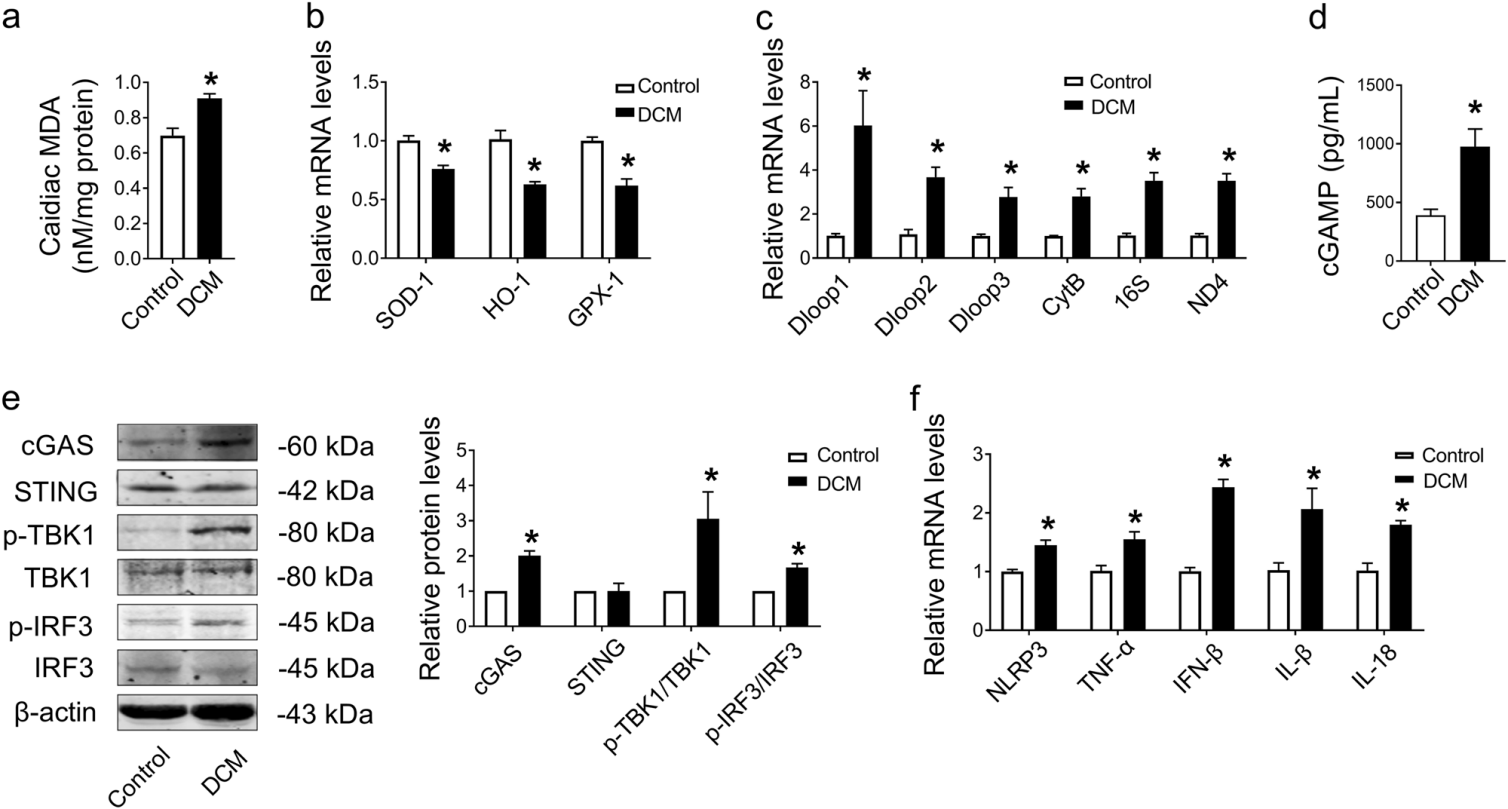
Oxidative stress-induced leakage of mtDNA and activation of the cGAS-STING pathway accompanied with inflammation in DCM mice hearts. **a** Assessment of the level of MDA in fresh heart tissues. *n* = 3 in each group. **b** mRNA levels of SOD-1, HO-1, and GPX-1 determined in tissues by qRT–PCR. *n* = 3 in each group. **c** mRNA levels of Dloop1, Dloop2, Dloop3, CytB, 16S, and ND4 determined in tissues by qRT–PCR. *n* = 3 in each group. **d** The contents of cGAMP in the hearts of DCM mice. *n* = 7 in each group. **e** Representative protein levels of cGAS, STING, p-TBK1/TBK1, and p-IRF3/IRF3 normalized to β-actin in heart tissues by western blot. *n* = 3 in each group. **f** mRNA levels of NLRP3, TNF-α, IFN-β, IL-1β, and IL-18 in tissues. *n* = 3 in each group. The results were normalized to β-actin. Values are the mean ± SEM. *P < 0.05 vs. the control group. DCM diabetic cardiomyopathy, MDA malondialdehyde.

Excessive oxidative stress is an initiator of DCM in response to disordered lipid metabolism and mitochondrial dysfunction [29]. We found that cardiac MDA, a marker of oxidative stress, was increased in the hearts of diabetic mice (Fig. 1a), which was accompanied by decreased mRNA levels of antioxidant markers (SOD-1, HO-1, and GPX-1) (Fig. 1b). Cytoplasmic mtDNA is a potent damage-associated molecular pattern (DAMP) in sterile inflammation following oxidative damage by the accumulation of mitochondrial ROS (mtROS) [30]. Hence, we detected the levels of mtDNA (Dloop1, Dloop2, Dloop3, CytB, 16S, and ND4) in the cytosol and found that all of these mtDNAs were upregulated in the cytosol of DCM mice compared with the control group (Fig. 1c), indicating the escape of mtDNA into the cytosol.

In parallel with the heightened levels of cytoplasmic mtDNA in the hearts of DCM mice, we next identified the key cytosolic dsDNA sensor and found that the cGAS-STING signaling pathway was activated as indicated by upregulated content of cGAMP in the hearts of DCM mice [31] (Fig. 1d), accompanied with the increased protein level of cGAS, phosphorylation of TANK-binding kinase 1 (TBK1) and type-I interferon (IFN) regulatory factor 3 (IRF3) in DCM mouse hearts compared with control mouse hearts, while the expression of STING showed no significant change (Fig. 1e). Furthermore, the mRNA levels of proinflammatory cytokines (NLRP3, TNF-α, IFN-β, IL-1β, and IL-18) were also elevated in DCM mouse hearts compared with control mouse hearts (Fig. 1f). These results demonstrated that the escape of mtDNA into the cytosol may trigger activation of the cGAS-STING signaling pathway, hence causing an inflammatory response in the hearts of DCM mice.

### STING deficiency improved cardiac function and suppressed cardiac hypertrophy in DCM mice

**Fig. 2:**
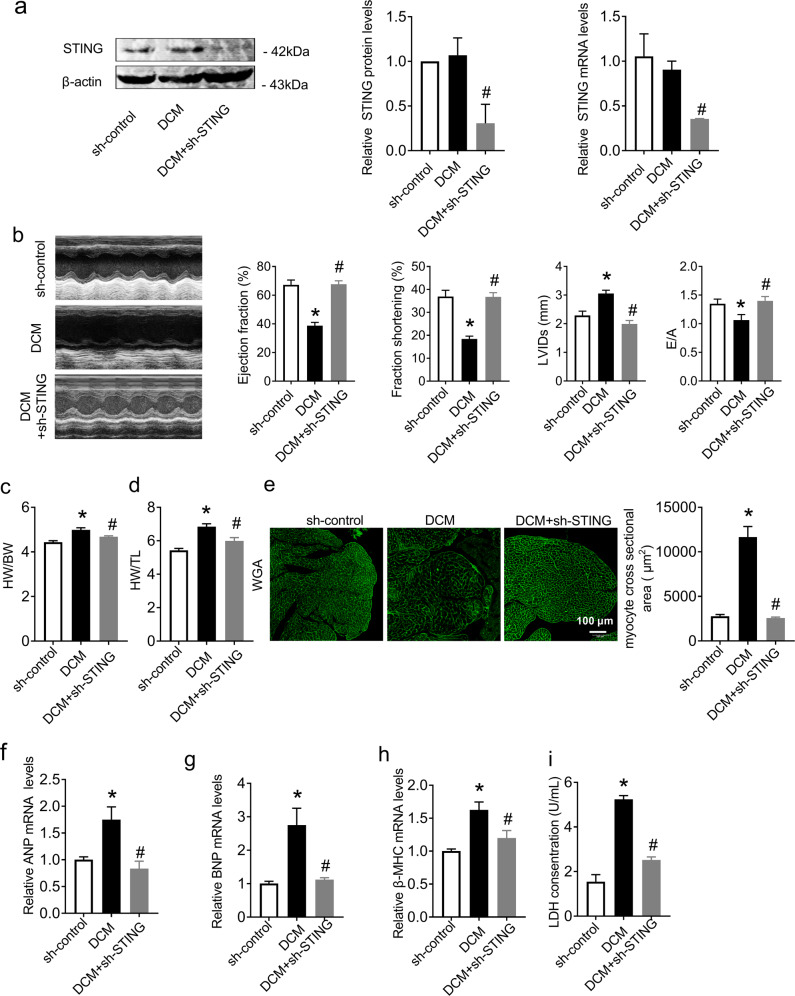
Knockdown of STING significantly improved cardiac function and inhibited cardiac hypertrophy in DCM mice. **a** Protein and mRNA levels of STING normalized to β-actin in heart tissues. *n* = 3 in each group. **b** Transthoracic echocardiography was performed to observe changes in cardiac function and morphology in both sh-control and DCM animals with or without shRNA of STING. Statistics of ejection fraction, fractional shortening, LVIDs and peak E to peak A (E/A) ratio. *n* = 7 in each group. **c**, **d** Statistical results of heart weight/body weight (HW/BW) and heart weight/tibia length (HW/TL). *n* = 6 in each group. **e** Quantitative analysis of myocyte cross-sectional area by WGA staining. *n* = 3 in each group. **f**–**h** mRNA levels of ANP, BNP, and β-MHC in heart tissues. *n* = 3 in each group. **i** LDH release in each group. *n* = 6 in each group. Values are the mean ± SEM. *P < 0.05 vs. the sh-control group, ^#^*P* < 0.05 vs. the DCM group. DCM diabetic cardiomyopathy, sh-STING shRNA of STING.

To evaluate the role of the cGAS-STING signaling pathway in cardiac function, we specifically knocked down cGAS and STING in the hearts of DCM mice by tail intravenous injection with AAV9 respectively. As shown in Figs. S1a and 2a, compared with DCM mice, the protein levels of cGAS and STING were decreased in the hearts of DCM mice after AAV9 injection. (Fig. S1a in Additional File 1 and Fig. 2a). Then, we evaluated the cardiac structure and function of mice by echocardiography. The results showed that the cardiac function of DCM mice was decreased, as evidenced by the decreased ejection fraction, fraction shortening, E/A ratio and increased left ventricle internal dimension in systole (LVIDs), while cGAS and STING knockdown both significantly improved cardiac function in DCM mice (Fig. S1b in Additional File 1 and 2b). We also found that STING deficiency reversed the increased heart weight/body weight ratio (HW/BW) and heart weight/tibia length ratio (HW/TL) in DCM mice (Fig. 2c, d), which indicated that knockdown of STING in the hearts of DCM mice had the potential to inhibit cardiac hypertrophy. Therefore, we assessed cardiac hypertrophy by wheat germ agglutinin (WGA) staining and detected the expression of hypertrophy-related genes (ANP, BNP, and β-MHC). The results showed that the myocyte cross-sectional area was increased in the hearts of DCM mice, with elevated mRNA levels of ANP, BNP and β-MHC, while STING knockdown alleviated these phenomena, indicating STING deficiency dramatically inhibited the deterioration of cardiac hypertrophy in DCM mice (Fig. 2e–h). Then, we detected the amount of LDH released in the serum of mice, which is considered a sensitive biomarker of cardiac injury. As shown in Fig. 2i, STING knockdown remarkably restored the elevated serum LDH level in DCM mice, suggesting an improvement in myocardial damage (Fig. 2i).

### STING deficiency inhibited cardiomyocyte pyroptosis and the inflammatory response in the hearts of DCM mice

**Fig. 3:**
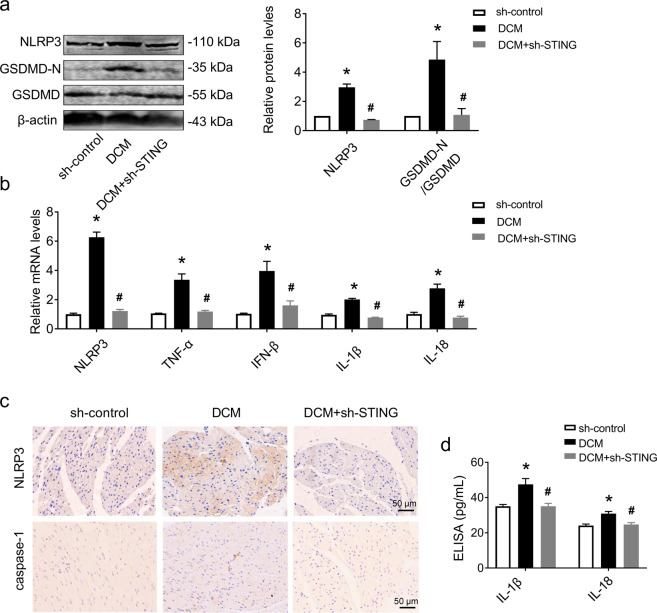
Knockdown of STING in DCM mice attenuated cardiomyocyte pyroptosis and inflammation. **a** Representative western blot analysis of NLRP3 and GSDMD-N/GSDMD in sh-control and DCM hearts after treatment with or without shRNA of STING and normalized to β-actin. *n* = 3 in each group. **b** mRNA levels of NLRP3, TNF-α, IFN-β, IL-1β, and IL-18 in heart tissues. *n* = 3 in each group. **c** Immunohistochemical staining analysis of NLRP3 and caspase-1 in heart tissues. *n* = 3 in each group. **d** Levels of IL-1β and IL-18 in serum analyzed by ELISA. *n* = 5 in each group. Values are the mean ± SEM. *P < 0.05 vs. the sh-control group, ^#^*P* < 0.05 vs. the DCM group. DCM diabetic cardiomyopathy, shRNA of STING, adeno-associated virus 9-sh-STING.

Previous studies have suggested that pyroptosis and inflammation are the hallmarks of cardiac hypertrophy [32, 33]. Given the role of the cGAS-STING signaling pathway in cardiac hypertrophy, we evaluated pyroptosis mediator proteins and proinflammatory cytokines in the hearts of DCM mice to identify whether cGAS-STING signaling pathway activation contributes to cardiomyocyte pyroptosis and the inflammatory response in DCM hearts. Compared with DCM mice, the protein levels of NLRP3 and GSDMD-N/GSDMD were decreased in the hearts of DCM + sh-STING mice, which was accompanied by decreased mRNA levels of NLRP3, TNF-α, IFN-β, IL-1β, and IL-18 (Fig. 3a, b). Immunohistochemical staining of NLRP3 and caspase-1 also showed that knockdown of STING effectively inhibited the expression of NLRP3 and caspase-1 (Fig. 3c) in the hearts of DCM mice. In addition, STING deficiency reduced the concentrations of IL-1β and IL-18 in serum, as evaluated by ELISA kits (Fig. 3d). We obtained the same results in DCM mice treated with Ru.521 or C176-1 (Fig. S1c in Additional File 1). These data suggested that STING knockdown inhibited cardiomyocyte pyroptosis and the inflammatory response in the hearts of DCM mice.

### Palmitic acid (PA) caused mtDNA escape into the cytosol and triggered cGAS-STING pathway activation in vitro

**Fig. 4:**
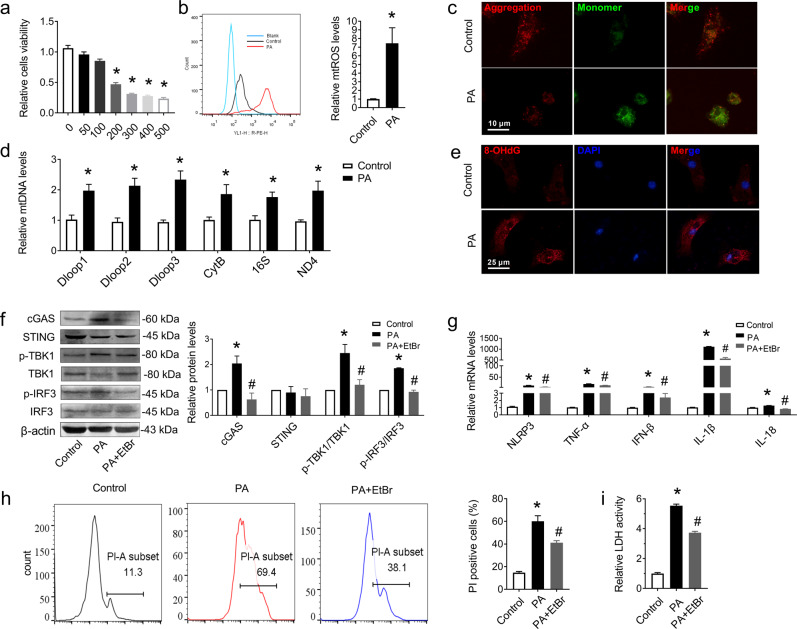
PA activated cGAS-STING pathway in cardiomyocyte by induced mtDNA release and oxidative damage, leading to cell death. **a** Effect of PA on cardiomyocyte viability. H9C2 cells were incubated with different concentrations (50, 100, 200, 300, 400, or 500 μM) of PA for 24 h and then assayed by cell counting kit-8. *n* = 5 in each group. **b** Representative flow cytometry image and quantitative analysis of the intracellular mtROS levels. *n* = 5 in each group. **c** Representative images of tetrachloro-tetraethyl benzimidazole carbocyanine iodide (JC-1) staining in NRCMs. *n* = 5 in each group. **d** mtDNA levels of Dloop1, Dloop2, Dloop3, CytB, 16S, and ND4 after treatment with or without PA in NRCMs. *n* = 5 in each group. **e** Oxidative guanine base damage of DNA was detected by fluorescence-based immunocytochemistry using specific antibodies for 8-OHdG in NRCMs. *n* = 6 in each group. **f** Western blot bands and analysis of cGAS, STING, p-TBK1/TBK1, and p-IRF3/IRF3 after treatment with PA with or without EtBr in H9C2 cells normalized to β-actin. *n* = 3 in each group. **g** The mRNA levels of NLRP3, TNF-α, IFN-β, IL-1β, and IL-18 in H9C2 cells. *n* = 3 in each group. **h** Representative flow cytometry image and the corresponding quantification showing the number of PI^+^ cells in the pyroptosis population. Hoechst 33342/PI double staining was performed by flow cytometry to detect pyroptosis in H9C2 cells. *n* = 6 in each group. **i** LDH release from each group of H9C2 cells. *n* = 9 in each group. Values are the mean ± SEM. *P < 0.05 vs. the control group, ^#^*P* < 0.05 vs. PA-treated H9C2 cells. PA palmitic acid.

To advance our mechanistic investigation of the effects of lipid toxicity on the myocardium, palmitic acid (PA), one of the major FFAs in plasma, was used to mimic lipid overload in H9C2 cardiomyocytes. A CCK-8 assay showed notably decreased cell viability after stimulation with 400 μM PA for 24 h (Fig. 4a), so 400 μM PA was chosen as the final concentration for intervention. Then, we found that mtROS was elevated in PA-treated cells, as indicated by flow cytometry (Fig. 4b). In addition, PA caused a loss of mitochondrial membrane potential, as indicated by a reduction in JC-1 aggregates labeled with red fluorescence and an increase in JC-1 monomers labeled with green fluorescence (Fig. 4c). A previous study proved that mtROS generation results in oxidized mtDNA and its release into the cytosol, which increases mitochondrial 8-hydroxy-2-deoxyguanosine (8-OHdG), a marker of oxidative DNA damage in the cytosol of the cell [34]. Our investigation further demonstrated that the levels of cytosolic mtDNA increased in neonatal mouse cardiomyocytes (NMCMs) after PA treatment (Fig. 4d), accompanied by increased DNA oxidative damage, as shown by 8-OHdG staining (Fig. 4e). These data indicated that PA treatment dramatically aggravated mitochondrial oxidative stress and mitochondrial dysfunction.

To further identify whether the leakage of mtDNA induced by PA was correlated with the activation of the cGAS-STING signaling pathway, we treated H9C2 cells with ethidium bromide (EtBr). Chronic EtBr exposure results in a reduction in mtDNA in the cytosol [35]. As shown in Fig. 4f, cGAS-STING signaling pathway was activated as indicated by the increased levels of cGAS, p-TBK1/TBK1, and p-IRF3/IRF3 (Fig. 4f), as well as the increased levels of cGAMP in PA treated NMCMs, while all the changes were reversed after EtBr treatment (Fig. S2a in Additional File 1). Moreover, mtDNA depletion downregulated the mRNA levels of proinflammatory cytokines (NLRP3, TNF-α, IFN-β, IL-1β, and IL-18; Fig. 4g). It has been demonstrated that STING will move from the endoplasmic reticulum to the perinuclear Golgi apparatus when activated by cGAMP, then conducts the downstream signal by phosphorylating TBK1 and IRF3 [36, 37]. So, immunofluorescence staining was conducted here to further demonstrate the activation of STING. Results showed that STING shifted from cytoplasm to perinuclear after cGAMP stimulus (Fig. S2b in Additional File 1). During pyroptosis, pores can form in the cell membrane and lead to the release of cellular contents and the positive staining of dead cells, which can be determined by a LDH release assay and PI staining, respectively [38]. The results showed that EtBr treatment also inhibited the increased cell pyroptosis in PA-treated cells, as indicated by the decreased number of PI-positive cells and reduced LDH activity (Fig. 4h, i).

### cGAS-STING mediated pyroptosis and the inflammatory response in PA-treated cardiomyocytes

**Fig. 5:**
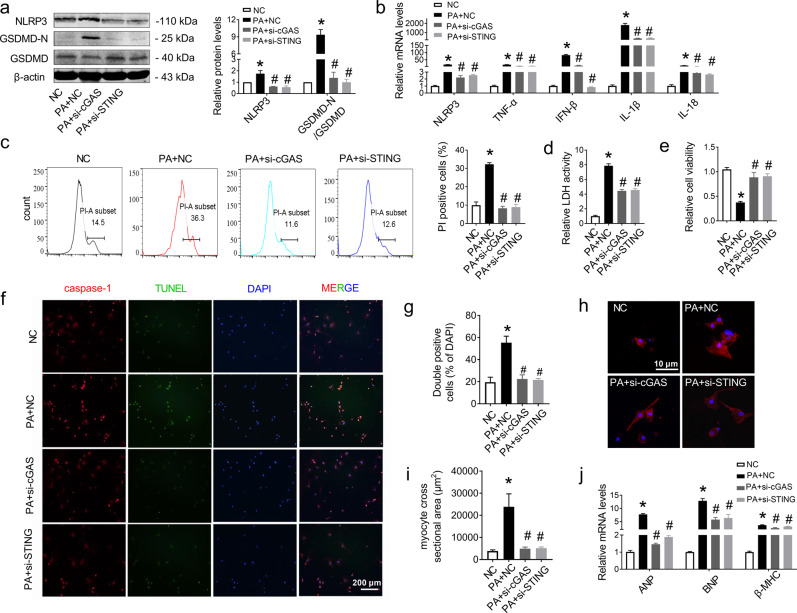
Inhibited cGAS-STING pathway effectively reduced cardiomyocyte pyroptosis and hypertrophy induced by PA treatment. **a** Western blot analysis of NLRP3 and GSDMD-N/GSDMD after treatment with PA with or without siRNA of cGAS or siRNA of STING in H9C2 cells normalized to β-actin. *n* = 3 in each group. **b** Relative mRNA levels of NLRP3, TNF-α, IFN-β, IL-1β, and IL-18 in H9C2 cells. *n* = 3 in each group. **c** Representative flow cytometry image and the corresponding quantification showing the number of PI^+^ cells as the pyroptosis population. *n* = 4 in each group. **d** LDH release from each group of H9C2 cells. *n* = 8 in each group. **e** H9C2 cells were incubated with PA with or without siRNA of cGAS or siRNA of STING for 24 h and then evaluated by cell counting kit-8. *n* = 8 in each group. **f**, **g** Representative images and quantitative analysis of caspase-1 (red) and TUNEL (green) double-positive cells in PA-induced NMCMs. *n* = 5 in each group. **h**, **i** Quantitative analysis of the myocyte cross-sectional area by α-actinin staining in NMCMs. *n* = 6 in each group. **j** mRNA levels of ANP, BNP, and β-MHC in H9C2 cells. *n* = 3 in each group. Values are the mean ± SEM. *P < 0.05 vs. NC, ^#^*P* < 0.05 vs. PA-treated cells. NC negative control, PA palmitic acid, si-cGAS small interfering RNA of cGAS, si-STING small interfering RNA of STING.

To further confirm the effect of the cGAS-STING signaling pathway on cell pyroptosis and the inflammatory response, we silenced cGAS or STING by transfecting small interfering RNAs (siRNAs). Previous studies have shown that PA can activate the NLRP3 inflammasome, hence activating caspase-1 to cleave both pro-IL-1β (31 kDa) into its 17 kDa bioactive form and pro-GSDMD (52 kDa) into GSDMD-N (31 kDa), which oligomerizes at the cell membrane to generate pores and cause cell pyroptosis [39]. Therefore, we detected the proteins that participated in pyroptosis and found that PA treatment upregulated the NLRP3 and GSDMD-N/GSDMD levels, and both siRNA-cGAS and siRNA-STING significantly rescued the increased expression of those proteins (Fig. 5a). Moreover, the mRNA levels of NLRP3, TNF-α, IFN-β, IL-1β, and IL-18 were upregulated in PA-treated cells, all of which were rescued by silencing cGAS or STING (Fig. 5b). A similar effect was found in Ru.521- or C176-1-treated H9C2 cells (Fig. S3a in Additional File 1). Further investigation showed that PA-induced pore formation, membrane rupture, and cell death, as indicated by the extensive PI-positive cell staining, increased LDH activity and decreased cell viability, which could be reversed by siRNA-cGAS or siRNA-STING (Fig. 5c–e). All of these results were consistent with those in Ru.521- or C176-1-treated cells (Fig. S3b–e in Additional File 1). To further characterize PA-induced pyroptosis in myocardial cells, we double-stained caspase-1 and TUNEL in NMCMs. The results in Fig. 5f, g show that both caspase-1 activity and the number of TUNEL-positive cells increased in PA-treated NMCMs. Silencing cGAS or STING prominently inhibited the activation of caspase-1 and reduced the number of TUNEL-positive cells induced by PA. In light of these results, we demonstrated that inhibition of the cGAS-STING signaling pathway effectively ameliorated pyroptosis and the inflammatory response induced by PA. Consistent with the in vivo data, siRNA-cGAS or siRNA-STING also inhibited the increased cardiomyocyte cross-sectional area induced by PA in NMCMs, as indicated by α-actinin staining, as well as the ANP, BNP, and β-MHC mRNA levels in H9C2 cells (Fig. 5h–j). These results demonstrated that the cGAS-STING pathway could mediate myocardial pyroptosis and the inflammatory response, which may promote cardiac hypertrophy in myocardial cells.

## Discussion

**Fig. 6:**
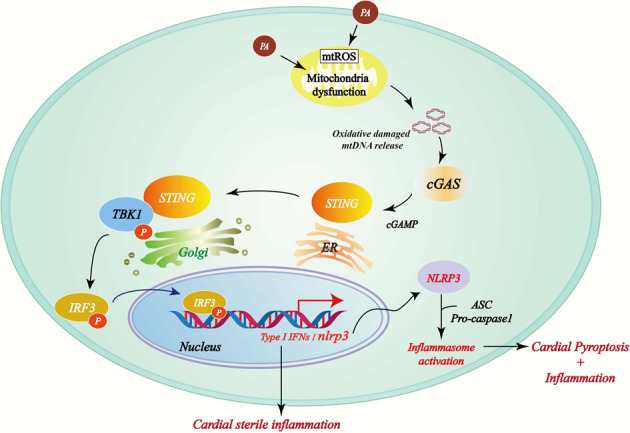
Proposed mechanism of PA-induced myocardial pyroptosis mediated by cGAS-STING pathway activation in DCM. PA causes mitochondrial damage and the release of mtDNA into the cytosol, which is converted to cGAMP by cGAS. cGAMP activates STING, which facilitates TBK-1-mediated IRF3 phosphorylation. Phosphorylated IRF3 enters the nucleus and induces type I interferon expression and NLRP3 inflammasome activation, which leads to the exacerbation of myocardial inflammation and pyroptosis.

In the current study, we have demonstrated that cGAS-STING signaling pathway was activated in the hearts of DCM mice, which was stimulated by the cytoplastic mtDNA raising from excessive accumulation of FFAs-induced lipotoxicity in the myocardium. Subsequently, cGAS-STING activated the NLRP3/caspase-1/GSDMD -mediated pyroptosis program and consequently triggered a series of inflammatory cascades. Moreover, cGAS-STING silencing effectively reduced myocardial pyroptosis and inflammation in DCM, further mitigated myocardial hypertrophy, and improved cardiac function (Fig. 6).

In our study, we found that the MDA content was significantly increased in the hearts of DCM mice, which was accompanied by lower levels of antioxidant mRNAs. A previous study demonstrated that mitochondria are more likely to be oxidatively damaged under these conditions. The fate of ROS-damaged mitochondria is either orderly disposal/recycling (mitophagy) or the pathologic release of mtDNA and cell death mediators [40]. We demonstrated that a large amount of mtDNA appeared in the cytoplasm of cardiomyocytes that were damaged by oxidative stress. Additionally, we found that the expression of the key DNA sensor protein cGAS in the cell was upregulated, which was accompanied by the increased cGAMP and phosphorylation and activation of its downstream effector proteins TBK1 and IRF3. Large numbers of proinflammatory factors were also released at the same time. Therefore, we speculated that oxidative stress could activate the cGAS-STING signaling pathway in the hearts of DCM mice and cause inflammation by inducing mtDNA escape into the cytosol. Next, to explore the role of the cGAS-STING pathway in cardiac function, we generated cardiac cGAS and STING-specific knockdown mice. The results showed that specific knockdown of both cGAS and STING in the heart can effectively improve heart function in DCM mice and inhibit myocardial hypertrophy and heart damage, proving that the cGAS-STING pathway has an important regulatory effect in the hearts of DCM mice after activation. To further clarify the molecular mechanism by which the cGAS-STING signaling pathway regulates DCM heart function, we focused on pyroptosis, a type of programmed cell death that is closely related to oxidative damage and plays an important role in the process of heart disease [41]. Our results showed that in the hearts of DCM mice, the levels of pyroptosis-related proteins and the release of proinflammatory factors were significantly reduced after STING knockdown.

In vitro experiments with PA were conducted in cardiomyocytes to simulate the lipotoxic conditions of cardiomyocytes, where excessive FFAs are taken up by DCM mice. Severe oxidative stress and mtDNA escape also appeared in cardiomyocytes after PA treatment, as indicated by the upregulated mtROS, decreased Δψm, increased cytoplasmic mtDNA, and increased 8-OHdG. Next, to further prove that the cGAS-STING signaling pathway could be activated by mtDNA escape, we introduced EtBr, a DNA depletion agent, into myocardial cells and found that the cGAS-STING signaling pathway was effectively inhibited, the inflammatory response was alleviated, and cell pyroptosis was decreased. To further identify the relationship between cGAS-STING and myocardial pyroptosis, we transfected cardiomyocytes with small interfering RNA to silence the cGAS and STING genes. The results showed that cGAS or STING knockdown not only downregulated the expression of pyroptosis-related proteins but also decreased the release of proinflammatory factors, reduced cardiomyocyte damage, lowered the number of pyroptotic cells, and increased cell viability. Moreover, silencing cGAS and STING reduced myocardial hypertrophy induced by PA treatment in vitro.

In summary, our study provides a new perspective for DCM treatment strategies. We proved that oxidative stress in cardiomyocytes could cause mitochondrial oxidative damage, leading to the release of mtDNA into the cytoplasm and further triggering cGAS-STING pathway activation. This mediates myocardial pyroptosis and the proinflammatory response, which eventually causes myocardial hypertrophy and promotes the progression of DCM. However, it has been proven that endoplasmic reticulum (ER) stress induced by lipotoxicity can also activate TBK1 and NF-κB (downstream of STING) by stimulating STING movement from the ER into the Golgi [42]; however, we did not discuss the role of ER stress in the activation of the cGAS-STING pathway here, which needs to be further studied to clarify the interaction between these two signaling axes.

## Conclusions

Our study demonstrated that mitochondrial oxidative damage and the escape of mtDNA into the cytosol caused by FFA-induced lipotoxicity could trigger the activation of the cGAS-STING pathway, which switches on the initiation of NLRP3 inflammasome-dependent pyroptosis and the proinflammatory response, hence promoting myocardial hypertrophy during the progression of DCM.

## Materials and methods

### Animals and treatments

Wild-type (WT) male C57BL/6J mice (4-week old) were purchased from LiaoNing ChangSheng Biotechnology Co., Ltd., China, and housed in pathogen-free animal houses. Mice were divided into three groups randomly: the sh-control group (*n* = 10), the DCM group (*n* = 10), and the DCM with myocardium-specific knockdown of STING (DCM + sh-STING) group (*n* = 10) or the DCM with myocardium-specific knockdown of cGAS (DCM + sh-cGAS) group (*n* = 10). Then, we induced myocardium-specific knockdown of cGAS or STING in mice by intravenous injection of adeno-associated virus 9 (AAV9) into the tail vein (cGAS-shRNA: CTGTGGATATAATTCTGGCTTCTCGAG

AAGCCAGAATTATATCCACAGTTTTT; STING-shRNA: GCATCAAGAATCGGG

TTTATTCTCGAGAATAAACCCGATTCTTGATGCTTTTTT). After 4 weeks, mice received five consecutive daily intraperitoneal injections of streptozotocin (STZ; 50 mg/kg, Sigma–Aldrich, St Louis, MO, USA) dissolved in 0.1 M cold sodium citrate buffer (pH 4.5) and were fed a high-fat diet to induce diabetes; sh-control mice received equivalent volume injections of citrate sodium buffer vehicle and were fed a normal diet. Mouse blood glucose levels were measured with a contour glucose meter (Roche, Germany) 3 days, 5 days, and 7 days after the injections, and the mice with blood glucose > 11.1 mmol/L in the tail vein were considered successful diabetic models. After 8 weeks, mouse cardiac function was evaluated with the Vevo2100 system.

To generate cGAS- or STING-inhibited DM mice, the C57BL/6J mice were divided into four groups randomly: the control group, the DCM group, the DCM injected with Ru.521 (DCM + Ru.521) group and the DCM injected with C176-1 (DCM + C176-1) group with *n* = 10 in each group. Mice received five consecutive daily intraperitoneal injections of streptozotocin (STZ; 50 mg/kg, Sigma–Aldrich, St Louis, MO, USA) dissolved in 0.1 M cold sodium citrate buffer (pH 4.5) and were fed a high-fat diet to induce diabetes. DM mice with blood glucose > 11.1 mmol/L were intraperitoneally injected with Ru.521 [43, 44] (an inhibitor of cGAS, 5 mg/kg, MedChemExpress, New Jersey, USA) or C176-1 [45] (an inhibitor of STING, 750 μM, 200 μL per mouse, MedChemExpress, New Jersey, USA) for 12 weeks. Normal mice were injected with corn oil as a control. No blinding was done to the researcher who conducted the measurements and outcome assessments. All animal studies were approved by the Animal Care Committee of Guangdong Pharmaceutical University (gdpulacspf2017277).

### Echocardiography

Mouse cardiac structure and function were evaluated by echocardiography with the Vevo2100 system (Fujifilm Visual Sonics, Toronto, Canada) at the Animal Center of Guangdong Pharmaceutical University. First, mice were anesthetized with 3% isoflurane and maintained under anesthesia with 1.5% isoflurane. Then, the cardiac function parameters, including ejection fraction (EF), fractional shortening (FS), left ventricle internal dimension in diastole (LVIDd), left ventricle internal dimension in systole (LVIDs) and E/A ratio, were measured.

### ELISAs of IL-1β and IL-18

The concentrations of the proinflammatory cytokines IL-1β and IL-18 in the serum of mice were determined by commercial ELISA kits (mice-sensitive kits for IL-1β and IL-18, Multi Sciences Biotech, Shanghai, China) according to the manufacturer’s instructions.

### Measurement of serum LDH levels

The lactic dehydrogenase (LDH) levels in serum were measured with a commercial lactic dehydrogenase assay kit (A020-1-2, Nanjing Jiancheng Biology Engineering Institute, Nanjing, Jiangsu, China). Briefly, the serum samples were first incubated with matrix buffer and coenzyme I for 15 min at 37 °C. Then, 2,4-dinitrophenylhydrazine was admixed with each sample and incubated for 15 min at 37 °C. Finally, 0.4 mmol/L NaOH was added to each mixture, and the absorbance was measured with a microplate reader (Bio‐Rad, Hercules, CA, USA) at 440 nm.

### WGA staining

Animal hearts were paraffin-embedded after fixation in 4% buffered formaldehyde for 24 h and sectioned into 5 µm thick slices. The cross-sectional areas of the cardiomyocytes in the left ventricle (LV) were observed by fluorescein-conjugated wheat germ agglutinin (WGA; 5 µg/mL, 25530, AAT Bioquest, USA) staining and evaluated by calculating the single myocyte cross-sectional areas measured by ImageJ software (National Institutes of Health, USA).

### Immunohistochemical analyses

Left ventricle tissue sections were incubated with anti-caspase-1 antibodies (WL02996a, Wanleibio, China, 1:200) and anti-NLRP3 antibodies (WL02635, Wanleibio, China, 1:500) overnight at 4 °C, followed by incubation with horseradish peroxide secondary antibodies (1:500, ab205718, Abcam, Cambridge, UK). Next, diaminobenzidine (DA1010, Solarbio, Beijing, China) was used to stain the tissue sections. Finally, the expression of caspase-1 and NLRP3 in the left ventricle was observed with a fluorescence microscope (Olympus DX51).

### Measurement of MDA levels

To assess the oxidative stress level, the contents of malondialdehyde (MDA) in the myocardium and H9C2 cells were detected using a commercial kit (S0131S, Beyotime, Shanghai, China) according to the instructions. Briefly, a 0.1 mL sample and 0.2 ml of MDA detection working solution were added to a centrifuge tube for determination. After mixing, the solution was placed in boiling water for 15 min. After cooling to room temperature, the sample was centrifuged at 1000 × *g* for 10 min at room temperature. Then, 200 µL of the supernatant was added to a 96-well plate, and the absorbance was measured at 532 nm with a microplate reader (Bio‐Rad, Hercules, CA, USA).

### Neonatal primary cardiomyocyte culture and treatment

Neonatal mouse cardiomyocytes (NMCMs) from 1 to 2 day old mouse heart tissues were isolated and cultured as previously described [33, 46]. H9C2 cells without mycoplasma contamination purchased from the Cell Bank of the Chinese Academy of Sciences (Shanghai, China) were used in subsequent experiments. The cells were cultured in normal DMEM (C11995500BT, Gibco, New York, USA) with 10% fetal bovine serum (ST30-3302, Gibco, New York, USA) and 1% penicillin–streptomycin solution (15140-122, Gibco, New York, USA). To knock down certain target genes, cells were incubated with cGAS small interfering RNA (siBDM1999A, siRNA-cGAS, RiboBio, Guangzhou, China) or STING siRNA (siBDM1999A, siRNA-STING, RiboBio, Guangzhou, China). The NMCMs and H9C2 cells in this study were all transfected with ribo FECTTM CP (C10511-05, Ribobio, Guangzhou, China) following the manufacturer’s instructions. When the cells reached 75% confluence, they were cultured with PA (400 µM) to construct a model of cardiomyocyte lipid toxicity in vitro. After PA treatment for 24 h, the cardiomyocytes were harvested for protein detection, RNA analysis, immunofluorescence staining analysis and LDH release measurements. Samples in one experiment constituted an independent replicate, and each experiment in our study was repeated at least 3 times.

### Generation of mtDNA-deficient cells

To exhaust the cytosolic mtDNA in cardiomyocytes, NMCMs were cultured in DMEM supplemented with 10% FBS, 1% penicillin–streptomycin solution and 2.5 μg/mL ethidium bromide (EtBr; 1239-45-8, MACKLIN, Shanghai, China) for 24 h as previously described [47].

### cGAMP treaments

To activate STING in cardiomyocytes, NMCMs cells were treated 2 μg/mL cGAMP (HY-100564-A, MedChemExpress, USA) for 4 h as previous study conducted [48].

### cGAS and STING inhibitor treatment

RU.521 and C176-1 were dissolved in DMSO to generate 10 mM stock solutions. H9C2 cells were pretreated with 20 μM C176-1 for 1 h followed by PA treatment for 24 h or cotreatment with 10 μM Ru.521 and PA for 24 h; DMSO vehicle treatment was used for the control group. After 24 h of culture, the cells were collected for RNA analysis, cell death assays, and CCK-8 assays.

### Western blot analysis

Cells or tissues were harvested, and 90 μg of total protein per sample was separated by gel electrophoresis and transferred to a hybridization nitrocellulose membrane (HATF00010, Merck, Darmstadt, Germany). The membrane was incubated overnight with primary antibodies (Table 1) at 4 °C. Before incubation with the fluorescence-conjugated anti-rabbit IgG secondary antibody (1:8000, 926-32211, LI-COR Biosciences, NE, USA), the membranes were washed with phosphate-buffered saline Tween (PBST) three times. Western blot bands were analyzed with an Odyssey imaging system (LI-COR, Inc., Lincoln, NE, USA).

**Table 1:** Antibodies for western blot.

Anti-body	Company	Catalog Number	Dilution ratio
cGAS	Gene Tex	GTX02874	1:1000
STING	Gene Tex	GTX85266	1:1000
IRF3	Gene Tex	GTX54366	1:500
p-IRF3	Cell Signaling Technology	#29047	1:500
TBK1	Cell Signaling Technology	#38066 S	1:1000
p-TBK1	Cell Signaling Technology	#3504	1:1000
NLRP3	Cell Signaling Technology	#15101	1:1000
GSDMD	ABclonal Technology	A20728	1:500
GSDMD-N	ABclonal Technology	A17308	1:500
β-Actin	Gene Tex	GTX109639	1:3000

### Total RNA isolation and quantitative real-time PCR (qPCR)

According to the manufacturer’s protocol and a previous study [46], total RNA of cardiac tissues and myocardial cells was extracted by TRIzol (15596018, Invitrogen, USA) and reversed-transcribed using a ReverTra Ace qPCR RT kit (FSQ-101, Toyobo, Japan). cDNA was amplified and detected with an ABI 7500 fast system (Applied Biosystems, CA, USA). The primer sequences are shown in Table 2. The data were analyzed using the 2^−ΔΔCt^ method and normalized to β-actin.

**Table 2:** Primer sequences for real-time RT-PCR.

Species	Gene	Forward	Reverse
Mouse	IFN-β	GGATCCTCCACGCTGCGTTCC	CCGCCCTGTAGGTGAGGTTGA
	STING	AAGTCTCTGCAGTCTGTGAAG	TGTAGCTGATTGAACATTCGGA
	IL-1β	GCCTCGTGCTGTCGGACCCATAT	TCCTTTGAGGCCCAAGGCCACA
	IL-18	GACTCTTGCGTCAACTTCAAGG	CAGGCTGTCTTTTGTCAACGA
	TNF-α	CCCTCCTGGCCAACGGCATG	TCGGGGCAGCCTTGTCCCTT
	NLRP3	ATTACCCGCCCGAGA AAGG	TCG CAGCAA AGATCCACACAG
	SOD1	CCATCAGTATGGGGACAATACA	GGTCTCCAACATGCCTCTCT
	HO-1	GGAGAATGGCAAGAATGAAGA	CCGCAGGAAGGTAAAGAG
	GPX-1	GCAGAGGTCCAGAAGAATGG	AGCATCCACCCAAATGACAC
	ANP	AAGAACCTGCTAGACCACCTGGAG	TGCTTCCTCAGTCTGCTCACTCAG
	BNP	GGAAGTCCTAGCCAGTCTCCAGAG	GCCTTGGTCCTTCAAGAGCTGTC
	β-MHC	GCAAGACGGTGACTGTGAAGGAG	GGTTGACGGTGACGCAGAAGAG
	Dloop1	AATCTACCATCCTCCGTGAAACC	TCAGTTTAGCTACCCCCAAGTTTAA
	Dloop2	CCCTTCCCCATTTGGTCT	TGGTTTCACGGAGGATGG
	Dloop3	TCCTCCGTGAAACCAACAA	AGCGAGAAGAGGGGCATT
	CytB	GCTTTCCACTTCATCTTACCATTTA	TGTTGGGTTGTTTGATCCTG
	16s	CACTGCCTGCCCAGTGA	ATACCGCGGCCGTTAAA
	ND4	AACGGATCCACAGCCGTA	AGTCCTCGGGCCATGATT
	mt.β-actin	GTGACGTTGACATCCGTAAAGA	GCCGGACTCATCGTACTCC
Rat	NLRP3	GAGCTGGACCTCAGTG ACAATGC	ACCAATGCGAGATCCTG ACAACAC
	IFN-β	ACAGCTCTCCGTCCTCGTAT	CCTTAGAGTAGAAAGACCG
	IL-18	TGCTGGATGCTCTGTATGG	CAAGTAGGGCTGTGTTTGC
	IL-1β	CACCTCTCAAGCAGAGCACAG	GGGTTCCATGGTGAAGTCAAC
	TNF-α	CTTCCCAGACTGTTCCCTG	CTGGTAGTTTAGCTCCGTTT
	ANP	GGAAGTCAACCCGTCTCA	AGCCCTCAGTTTGCTTTT
	BNP	TTTGGGCAGAAGATAGACCG	AGAAGAGCCGCAGGCAGAG
	β-MHC	GTGAAGGGCATGAGGAAGAGT	AGGCCTTCACCTTCAGCTGC

### ELISA assays of cGAMP

The concentrations of the cGAMP in the tissue of mice and NMCMs were determined by the commercial ELISA kits (mice sensitive kit for cGAMP was purchased from Cayman Chemical, ML, USA) according to the manufacturer’s instructions.

### Immunofluorescence staining

NRCMs seeded on coverslips were first fixed with 4% formaldehyde and then permeabilized with 0.3% Triton X-100. Subsequently, the cells were blocked with 1% BSA/10% goat serum in PBS for 30 min at room temperature and incubated with an anti-STING antibody (1:500, GTX85266, Gene Tex, USA), an anti-caspase-1 antibody (1:500, WL02996a, Wanleibio, China), an anti-α-actinin antibody (1:200, A7811, Sigma, USA) and an anti-DNA/RNA damage antibody (1:100, ab62623, Abcam, UK) diluted in blocking buffer at 4 °C overnight. Then, to probe the proteins, Alexa Fluor 594-conjugated goat anti-rabbit or anti-mouse secondary antibodies (1:200, Thermo Fisher, Massachusetts, USA) were used.

### Cell death assay

For the detection of LDH release, the LDH activity in cell culture supernatants was detected using an LDH assay kit (C0017, Beyotime, Beijing, China) according to the manufacturer’s instructions. Pyroptotic cell death was evaluated by Hoechst 33342/PI staining (V13244, Thermo Fisher Scientific, Carlsbad, USA). For Hoechst 33342/PI staining, cells were collected and suspended in 1 mL of cold phosphate-buffered saline (PBS). Then, 1 mL of cell suspension was added to 1 μL of the Hoechst 33342 stock solution and 1 μL of the PI stock solution. After incubation on ice for 30 min, the cells were analyzed by flow cytometry. Dual excitation was at UV/488 nm, and fluorescence emission was set at ~460 nm and >575 nm.

### Mitochondrial membrane potential (Δψm) evaluation

Changes in mitochondrial membrane potential (Δψm) were detected with a mitochondrial staining kit (C2006, Beyotime, Shanghai, China) according to the manufacturer’s instructions. For this assay, treated NMCMs were incubated with 1 mL of 2 µmol/L JC-1 dye solution in DMEM at 37 °C for 20 min and then observed with an Olympus DX51 microscope.

### Isolation and detection of cytosolic mtDNA by qPCR

PA-treated NRCMs or heart tissue was lysed in cell lysis buffer (BioVision), and all samples were centrifuged at 700 × *g* at 4 °C for 10 min to remove the nuclei and intact cells. The supernatant was normalized to the same volume according to the protein concentration. To isolate the cytosolic fraction, the supernatant was further centrifuged at 10,000 × *g* for 30 min at 4 °C. Quantitative PCR was used to detect the levels of cytosolic mtDNA, and the sequences that coded for mtDNA were used as primers. The primer sequences are shown in Table 2. The data were analyzed using the 2^−ΔΔCt^ method and normalized to β-actin.

### Detection of mitochondrial ROS

To evaluate intracellular mtROS production, H9C2 cells were incubated with MitoSOX (M36008, Invitrogen, Carlsbad, CA). After incubation, the fluorescence intensity was recorded using a flow cytometer (Beckman Coulter, Cytoflex).

### CCK-8 assay

According to the manufacturer’s protocols, cell viability was analyzed by cell counting kit‐8 (CCK-8, C0038, Beyotime, Shanghai, China). H9C2 cells, which were seeded in 96‐well microplates (3599, Corning, USA) and cultured with 100 µL of DMEM, were treated with PA (400 µM) for 24 h. To assay cell viability, 10 μL of CCK‐8 reagent was added to each well followed by the culture at 37 °C for 2 h. The absorbance was measured at 450 nm using a microplate reader (Bio‐Rad, Hercules, CA, USA).

### TdT-mediated dUTP nick end labeling (TUNEL) staining

Double staining with TUNEL (green) and caspase-1 (red) was performed to quantify pyroptotic cell death 24 h after PA treatment. For TUNEL staining, an in situ cell death detection kit (52733700, Roche, USA) was used according to the manufacturer’s instructions. Data are expressed as the ratio of TUNEL and caspase-1 double‐positive cells (%).

### Statistical analyses

The sample size was chosen according to previous observations, which perform similar experiments to see significant results, or the results from our preliminary experiments. Each experiment was repeated three times. All data in our study are presented as the mean ± SEM and were analyzed by GraphPad Prism 7 software. Differences between two groups were analyzed by Student’s unpaired *t* test. Multiple comparisons among different groups were analyzed by one-way ANOVA followed by a post hoc Tukey’s test. Statistical significance was defined as *P* < 0.05. Normal distribution of data and data variation were not assessed.

## Supplementary information

Supplemental Material-full bands of WB

Additional File 1

figure S1

figure S2

figure S3

## Data Availability

All datasets generated for this study are included in the article/supplementary material.
