# Supplementary material for: Mitochondrial damage and activation of the cytosolic DNA sensor cGAS–STING pathway lead to cardiac pyroptosis and hypertrophy in diabetic cardiomyopathy mice
Source: Cell Death Discov. 2022 May 11;8:258. doi: 10.1038/s41420-022-01046-w (PMC9091247; doi:10.1038/s41420-022-01046-w)

**Mitochondrial Damage and Activation of the Cytosolic DNA Sensor cGAS–STING Pathway Lead to Cardiac Pyroptosis and Hypertrophy in Diabetic Cardiomyopathy Mice**

*Meiling Yan^1†^, Yun Li^1†^, Qingmao Luo^1†^, Wenru Zeng^1^, Xiaoqi Shao^1^, Lun Li^1^, Qing Wang^1^, Dongwei Wang^1^, Yue Zhang^1^, Hongtao Diao^1^, Xianglu Rong^2-5^, Yunlong Bai^6-7^ and Jiao Guo^2-5*^*

Full WB bands of cGAS, STING, pTBK1, TBK1, pIRF3 and IRF3 shown in Fig1e.


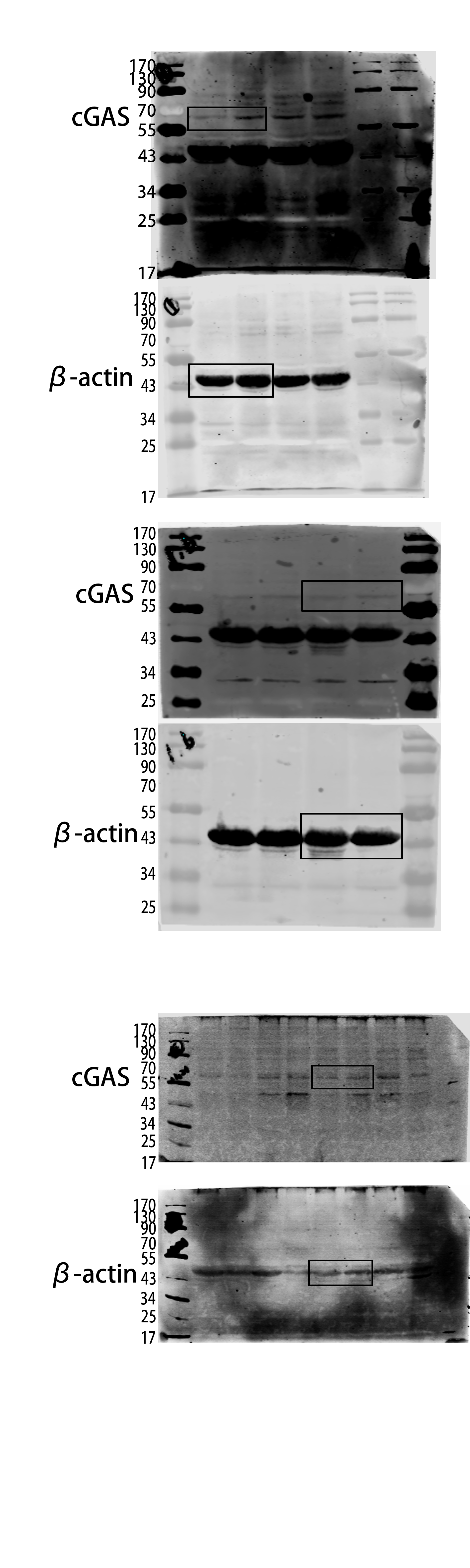


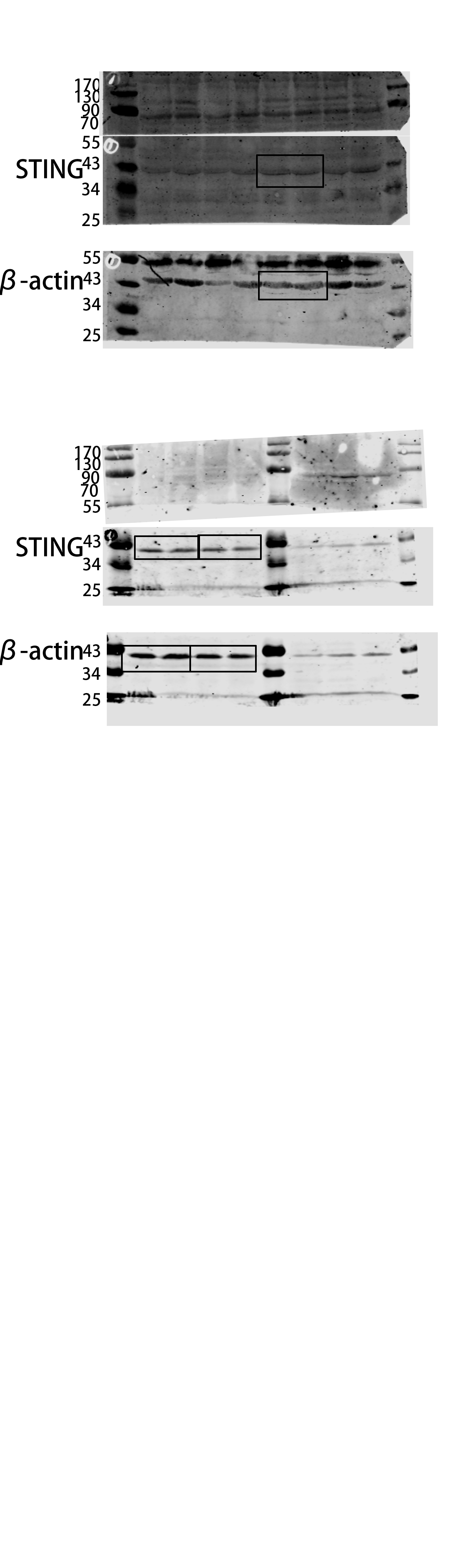


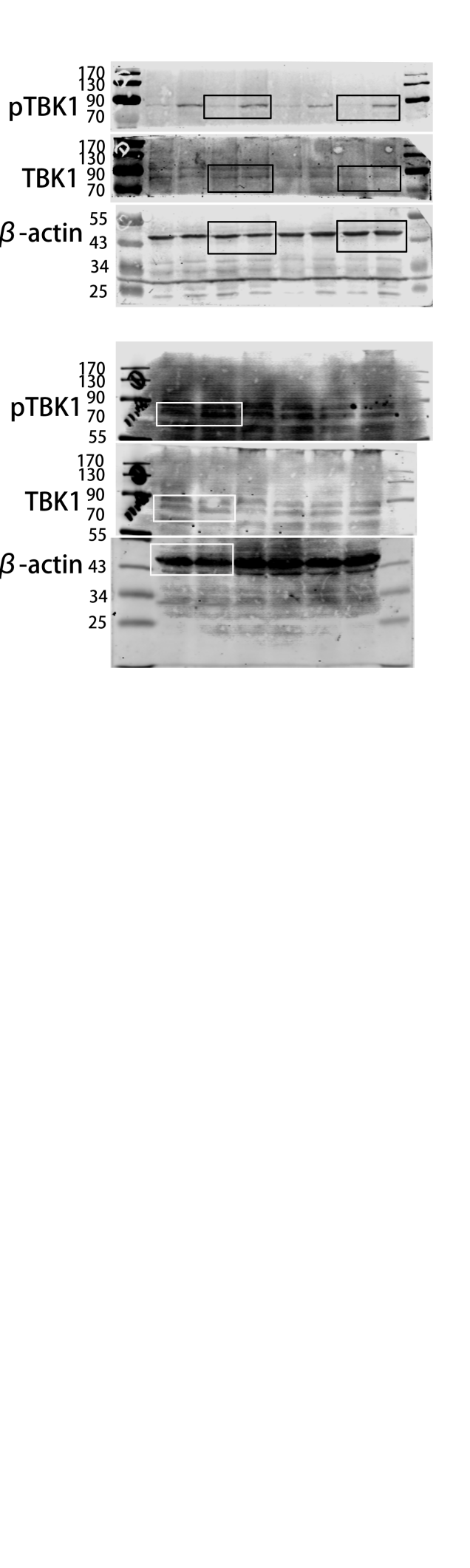


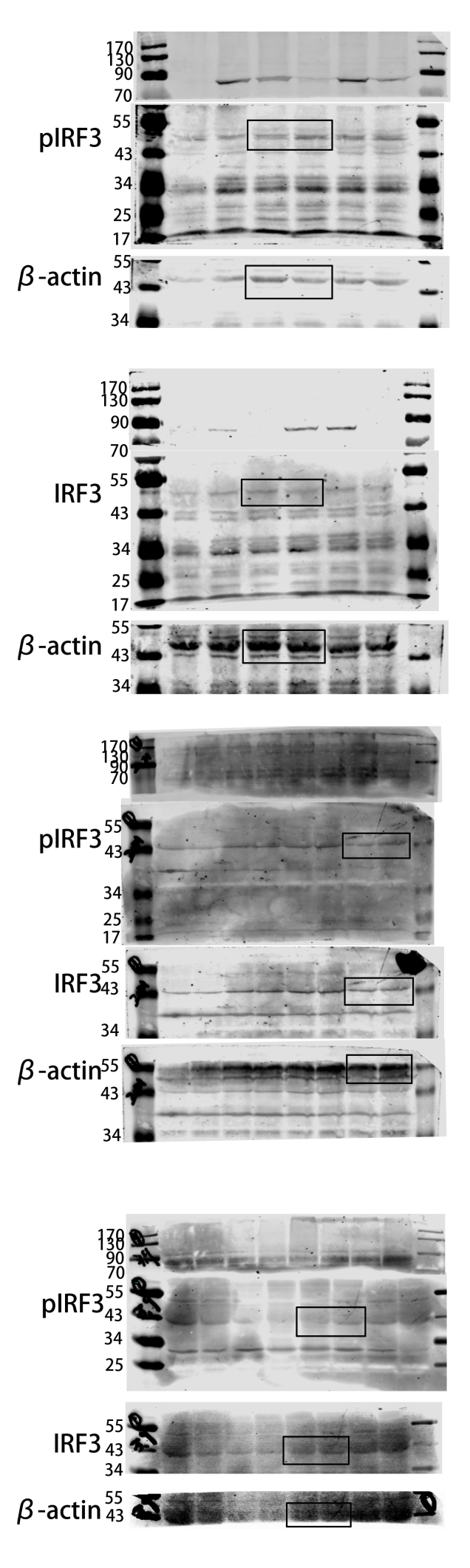


Full WB bands of STING shown in Fig2a.


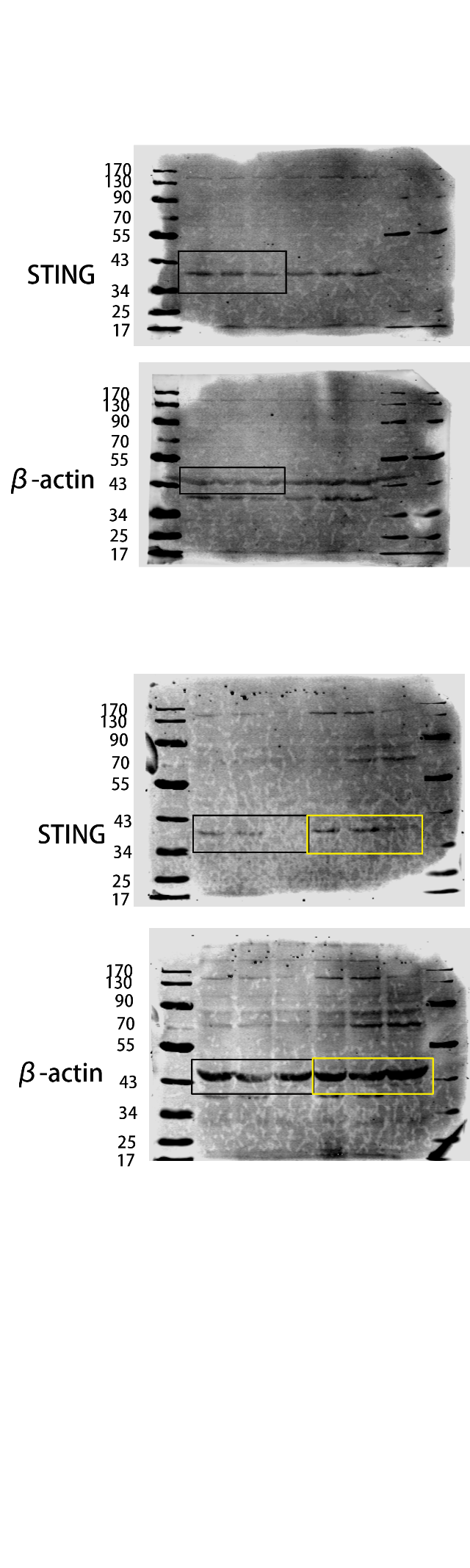


Full WB bands of NLRP3, GSDMD, GSDMD-N shown in Fig3a.


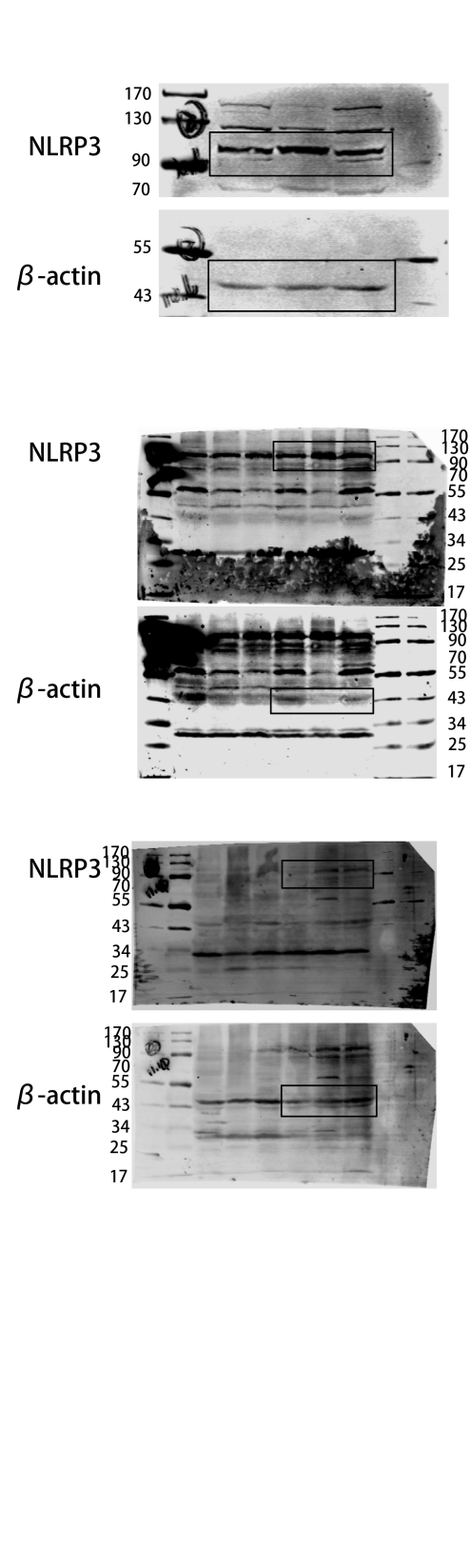


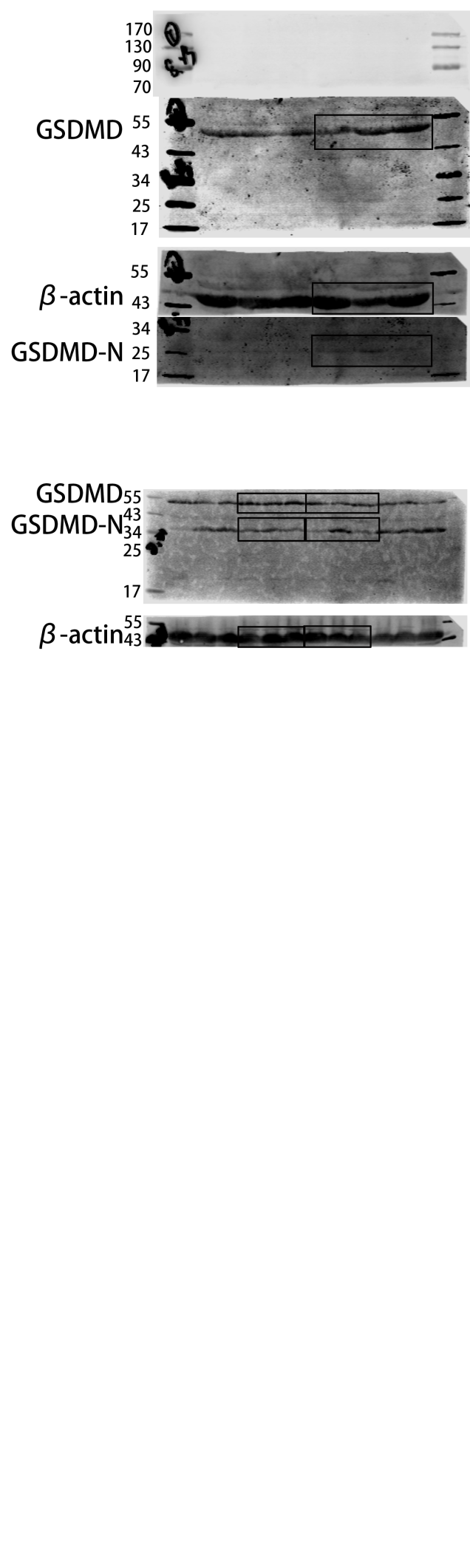


Full WB bands of cGAS, STING, pTBK1, TBK1, pIRF3 and IRF3 shown in Fig4f.


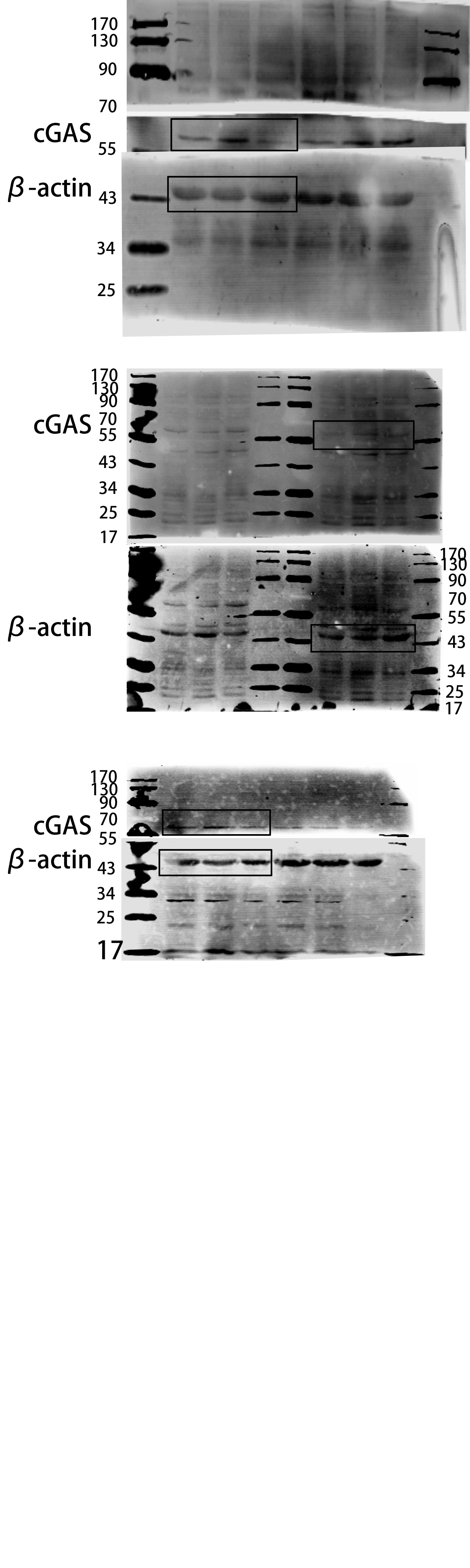


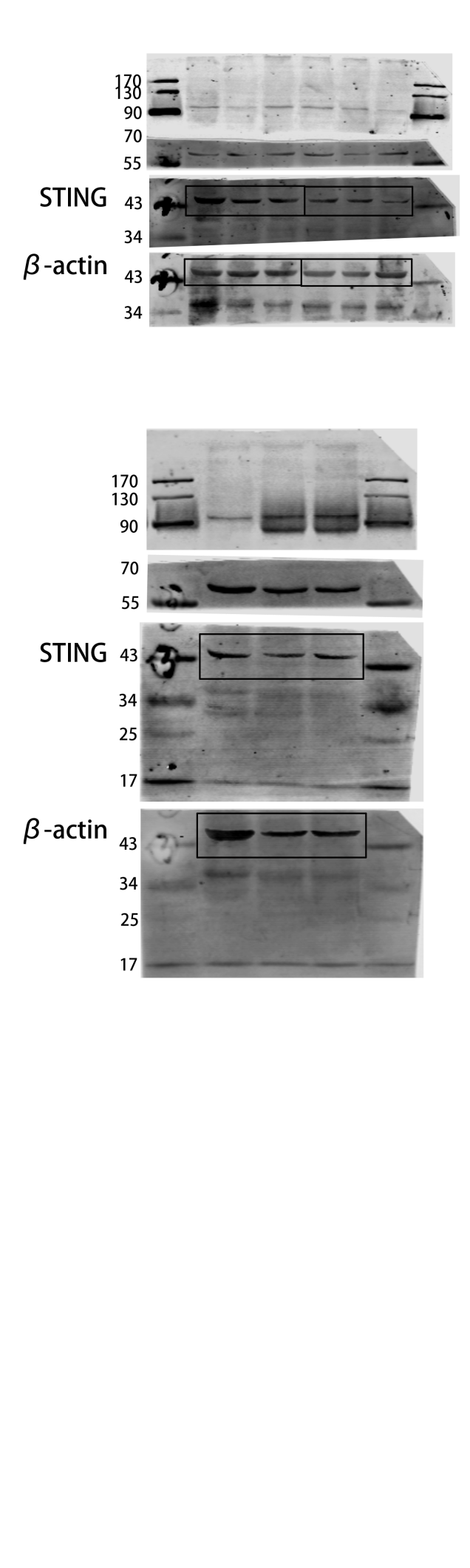


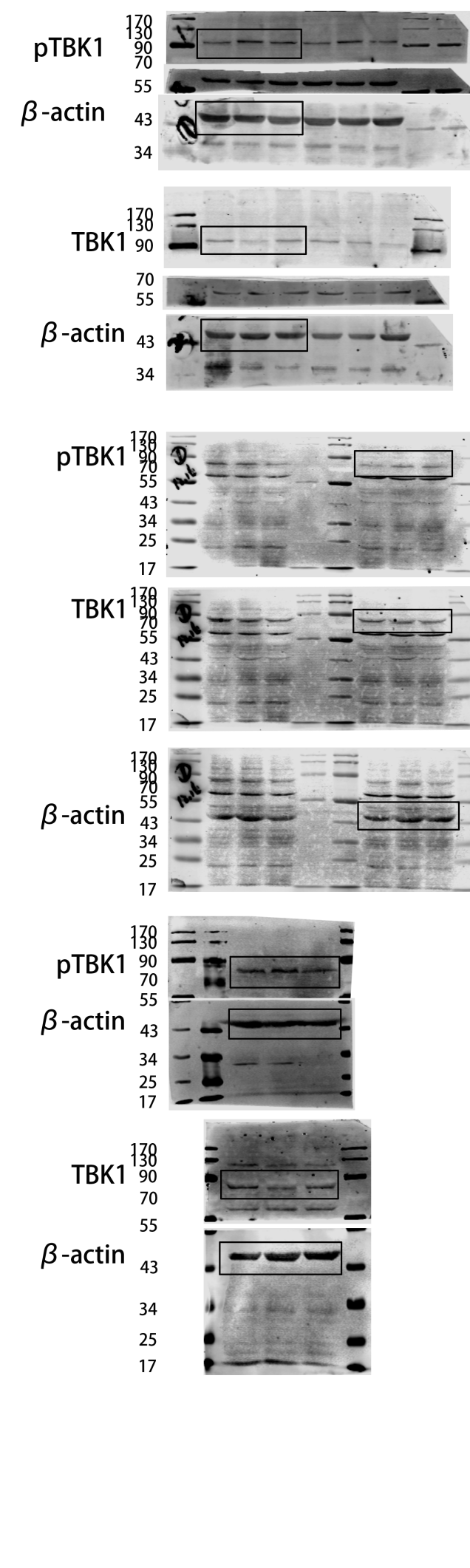


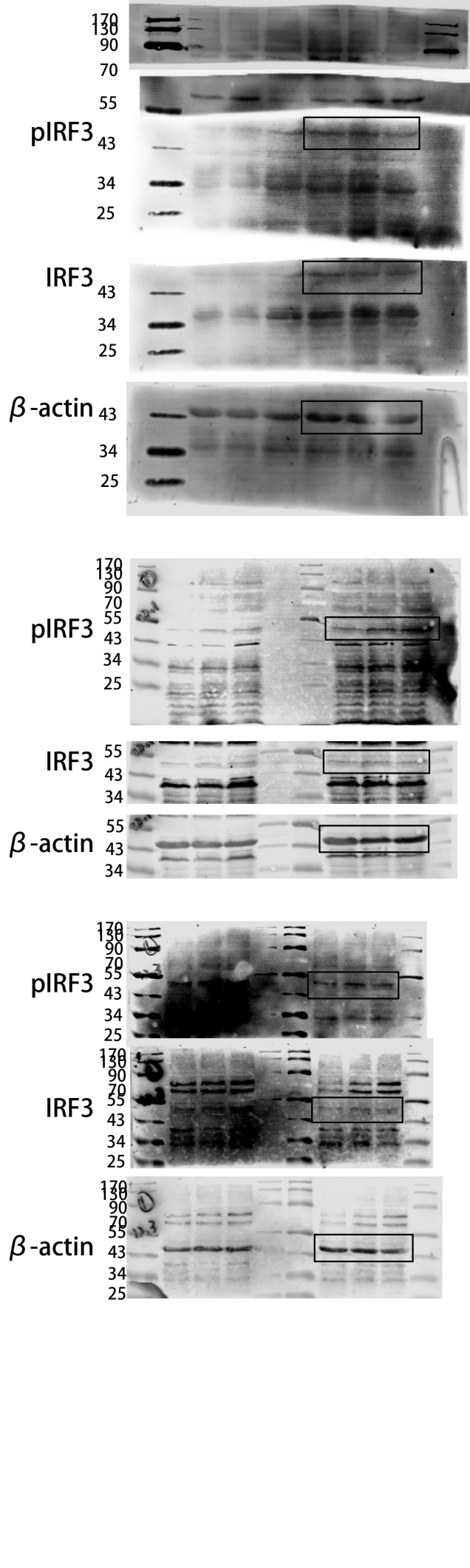


Full WB bands of NLRP3, GSDMD-N and GSDMD shown in Fig5a.


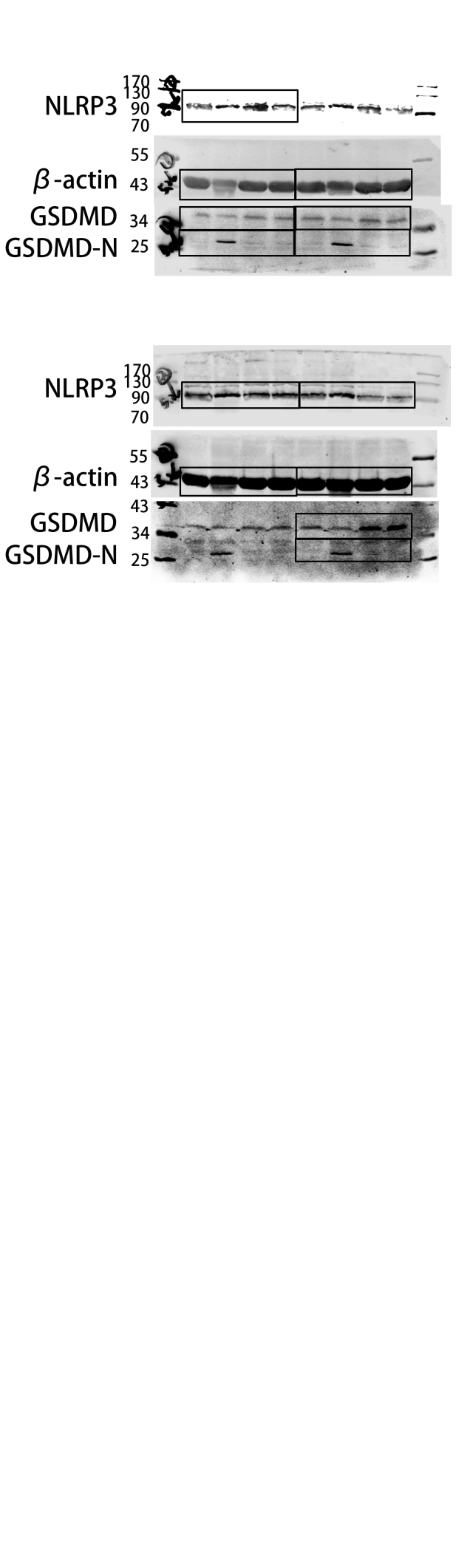


Full WB bands of cGAS shown in Fig S1a.


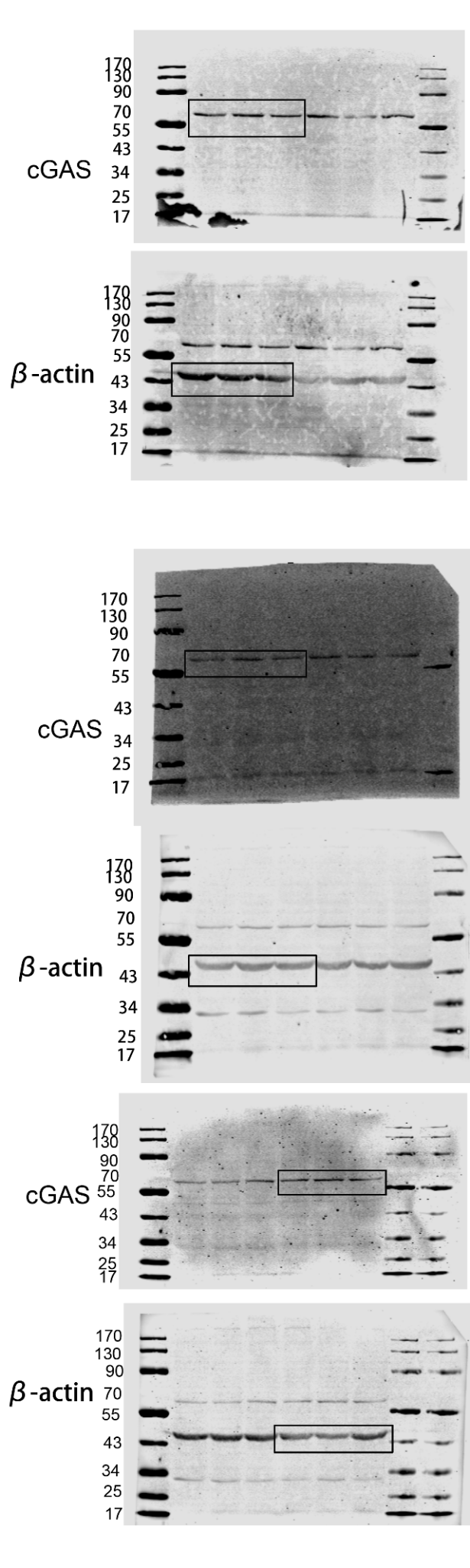

Supplement: Supplementary file 1 — Supplemental Material-full bands of WB [file 41420_2022_1046_MOESM1_ESM.docx]
