# Supplementary material for: Mitochondrial damage and activation of the cytosolic DNA sensor cGAS–STING pathway lead to cardiac pyroptosis and hypertrophy in diabetic cardiomyopathy mice
Source: Cell Death Discov. 2022 May 11;8:258. doi: 10.1038/s41420-022-01046-w (PMC9091247; doi:10.1038/s41420-022-01046-w)
Supplement: Supplementary file 2 — Additional File 1 [file 41420_2022_1046_MOESM2_ESM.docx]

**Mitochondrial Damage and Activation of the Cytosolic DNA Sensor cGAS–STING Pathway Lead to Cardiac Pyroptosis and Hypertrophy**

*Meiling Yan^1†^, Yun Li^1†^, Qingmao Luo^1†^, Wenru Zeng^1^, Xiaoqi Shao^1^, Lun Li^1^, Qing Wang^1^, Dongwei Wang^1^, Yue Zhang^1^, Hongtao Diao^1^, Xianglu Rong^2-5^, Yunlong Bai^6-7^* and Jiao Guo^2-5*^*


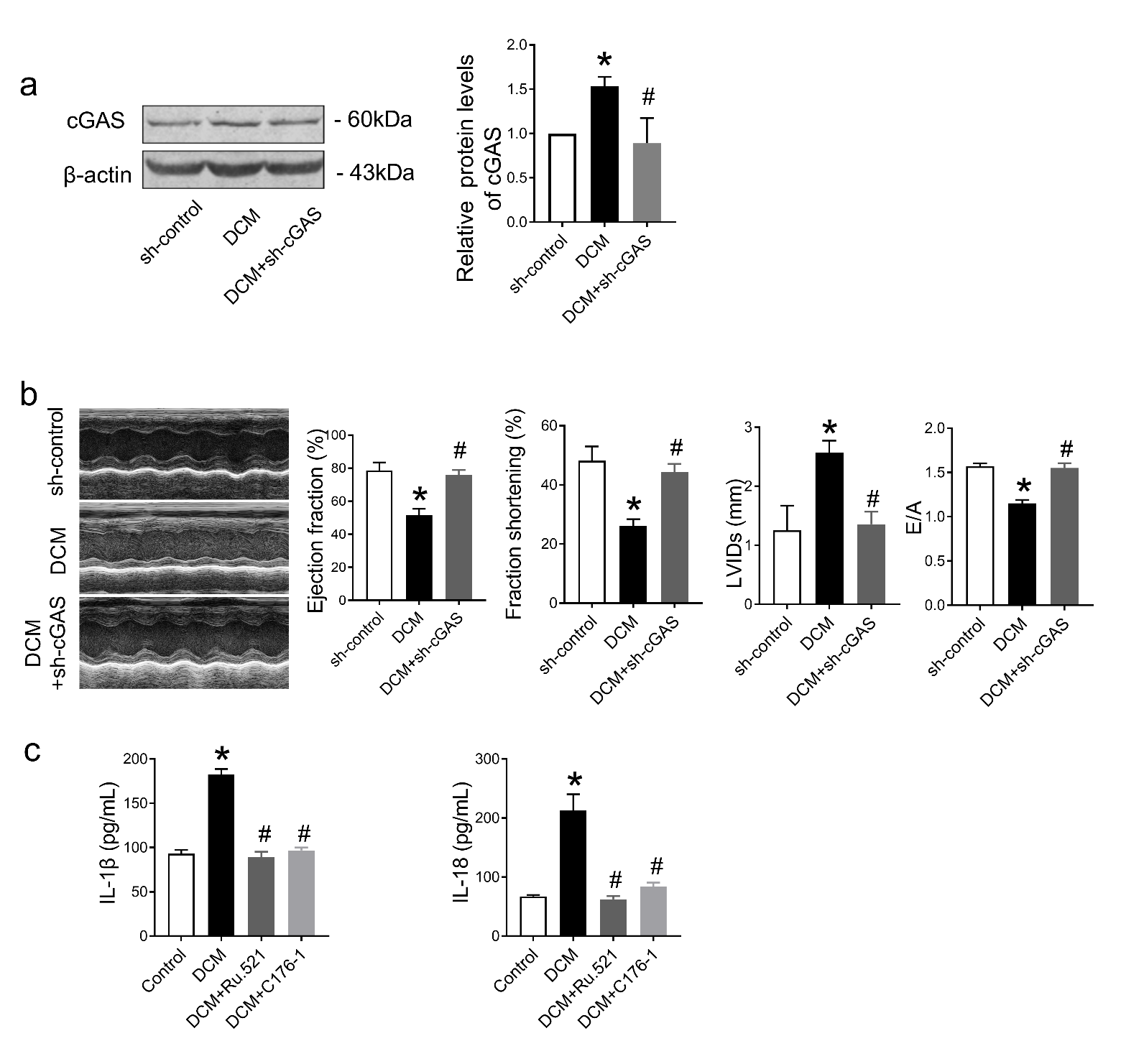


**Figure S1 | cGAS activity was increased in DCM mice heart tissue, while inhibited cGAS-STING pathway effectively rescued heart function and reduced inflammation in DCM mice.** **(a)** Representative protein levels of cGAS normalized to β-actin in the heart tissues by western blotting. n=3 in each group. **(b)** Transthoracic echocardiography was performed to observe changes in cardiac function and morphology in control and DCM animals with or without shRNA of cGAS. Statistics of ejection fraction, fractional shortening, LVIDs and peak E to peak A (E/A) ratio. n = 7 in each group. **(c)** The levels of IL-1β and IL-18 in serum were analyzed by ELISA. n=5 in each group. Values are mean ± SEM. **P* < 0.05 vs Control or sh-control group, #*P* < 0.05 vs DCM group. cGAMP, Cyclic GMP-AMP. DCM, diabetic cardiomyopathy. si-cGAS, siRNA of cGAS. Ru.521, inhibitor of cGAS. C176-1, inhibitor of STING.


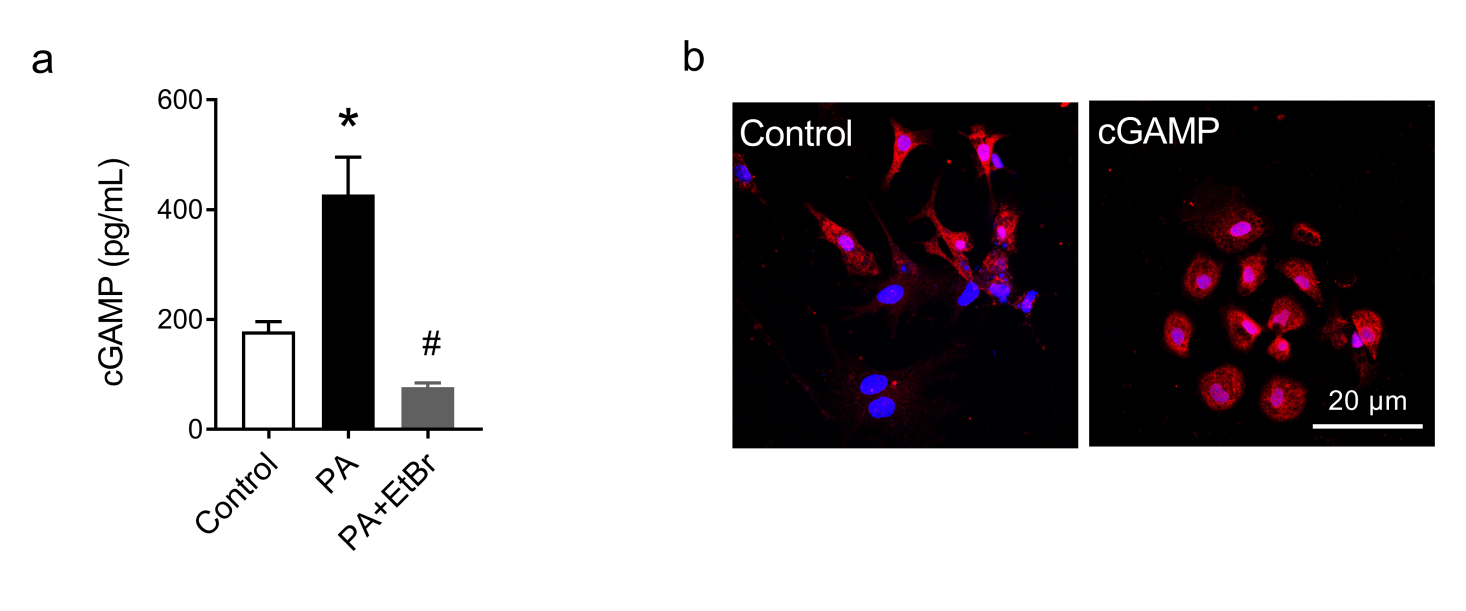


**Figure S2 | The content of cGAMP in NMCMs and the position changing of STING after cGAMP stimulus. (a)** The levels of cGAMP in NMCMs was analyzed by ELISA. n=7 in each group. **(b)** Representative images of STING in cGAMP treated NMCMs. n=5 in each group. Values are mean ± SEM. **P* < 0.05 vs Control group. cGAMP, cyclic GMP-AMP.


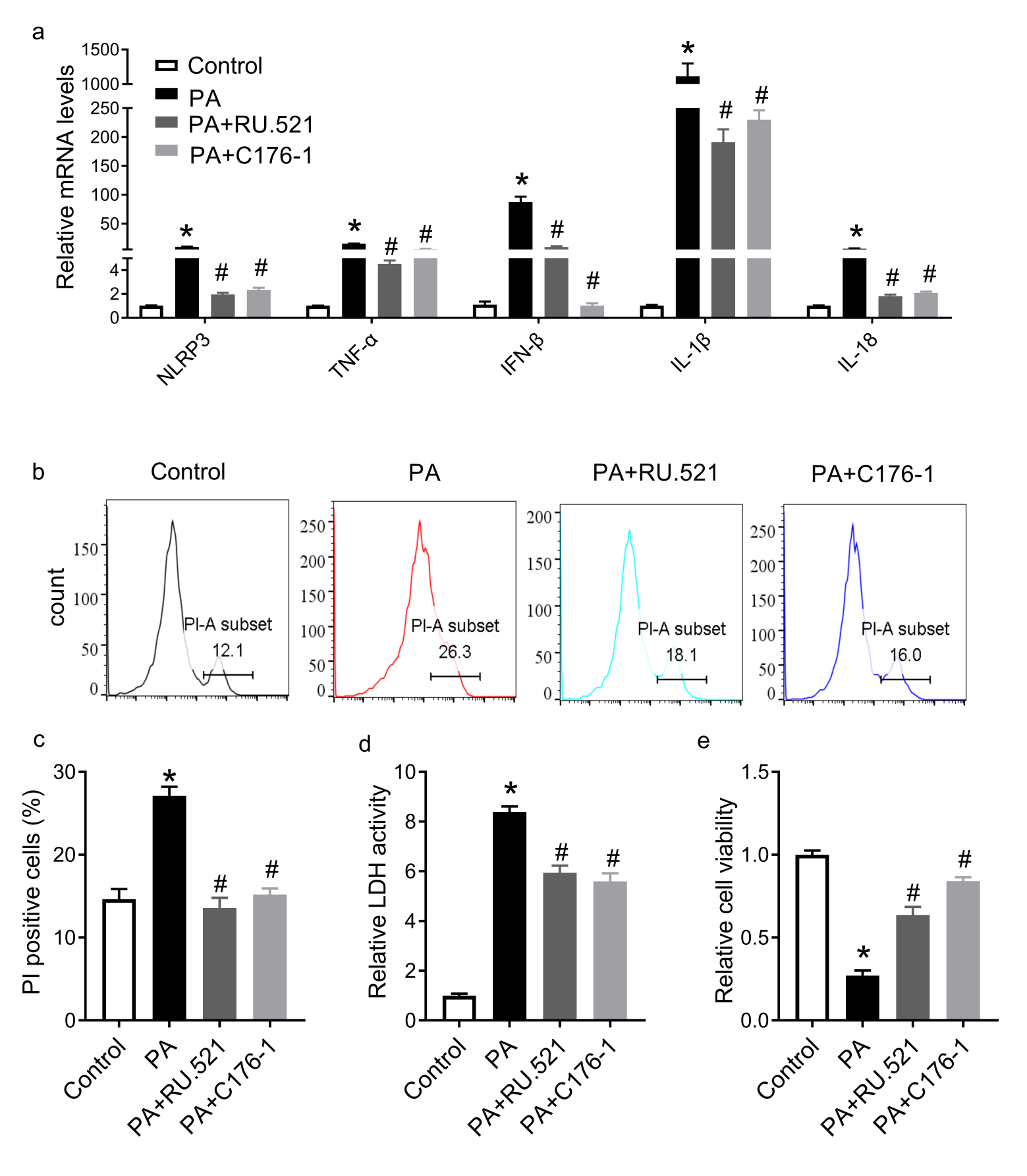


**Figure S3 | Ru.521 and C176-1 treatment improved cardiomyocyte inflammation and pyroptosis. (a)** Relative mRNA levels of NLRP3, TNF-α, IFN-β, IL-1β and IL-18 in H9C2 cells. n=6 in each group. **(b-c)** Representative flow cytometric image and the corresponding quantification showing PI^+^ cells as pyroptosis population. n=5 in each group. **(d)** LDH release in each group. n =7 in each group. **(e)** Cell viability detection. Cells were incubated with PA with or without Ru.521 or C176-1 for 24 h and then tested with cell counting kit-8 in H9C2 cells. n=10 in each group. Values are mean ± SEM. **P* < 0.05 vs control group, #*P* < 0.05 vs PA-treated H9C2 cells.
