# Supplementary figures and images for: Mitochondrial damage and activation of the cytosolic DNA sensor cGAS–STING pathway lead to cardiac pyroptosis and hypertrophy in diabetic cardiomyopathy mice
Source: Cell Death Discov. 2022 May 11;8:258. doi: 10.1038/s41420-022-01046-w (PMC9091247; doi:10.1038/s41420-022-01046-w)

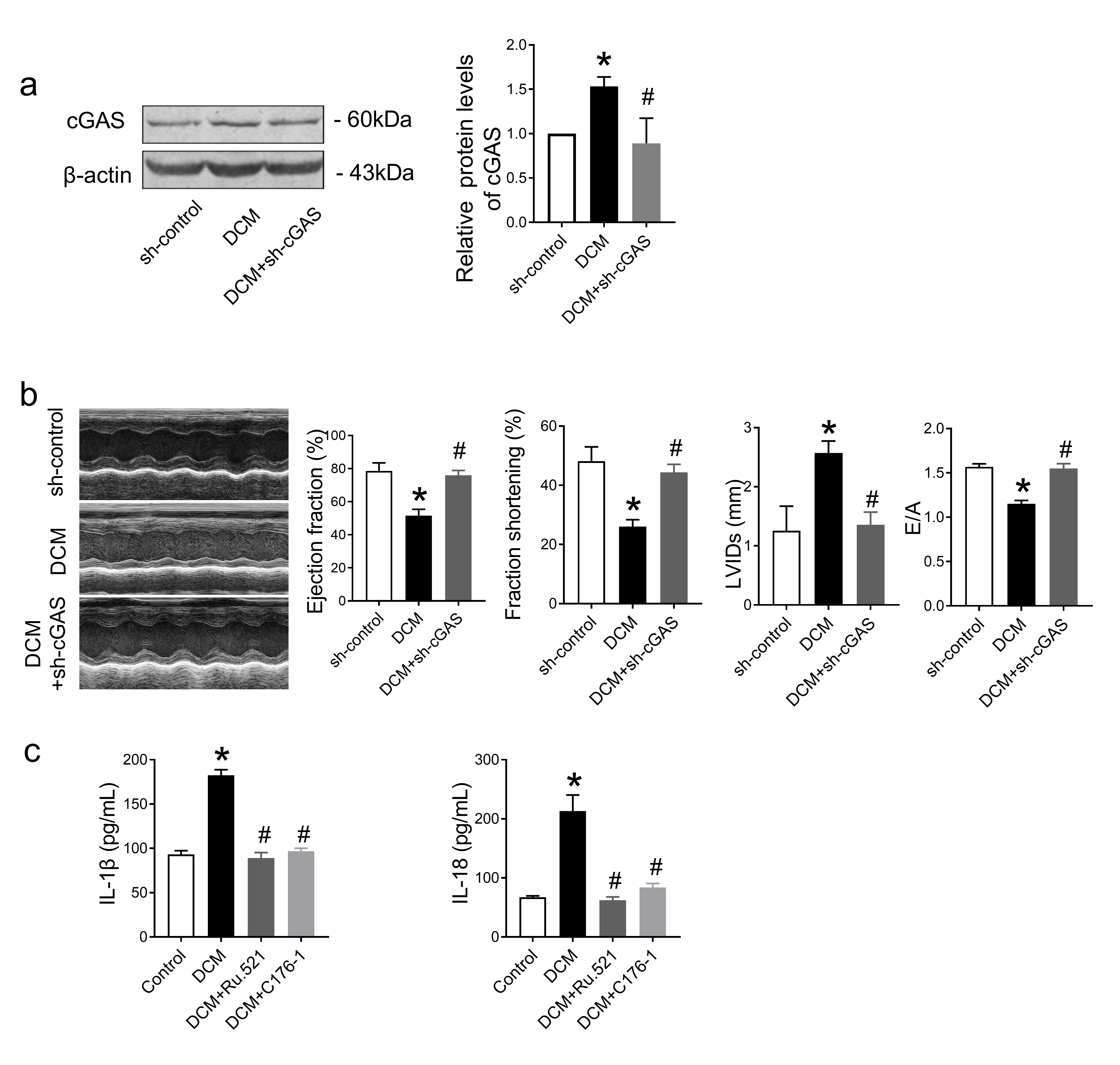

Supplement: Supplementary file 3 — figure S1 [file 41420_2022_1046_MOESM3_ESM.png]

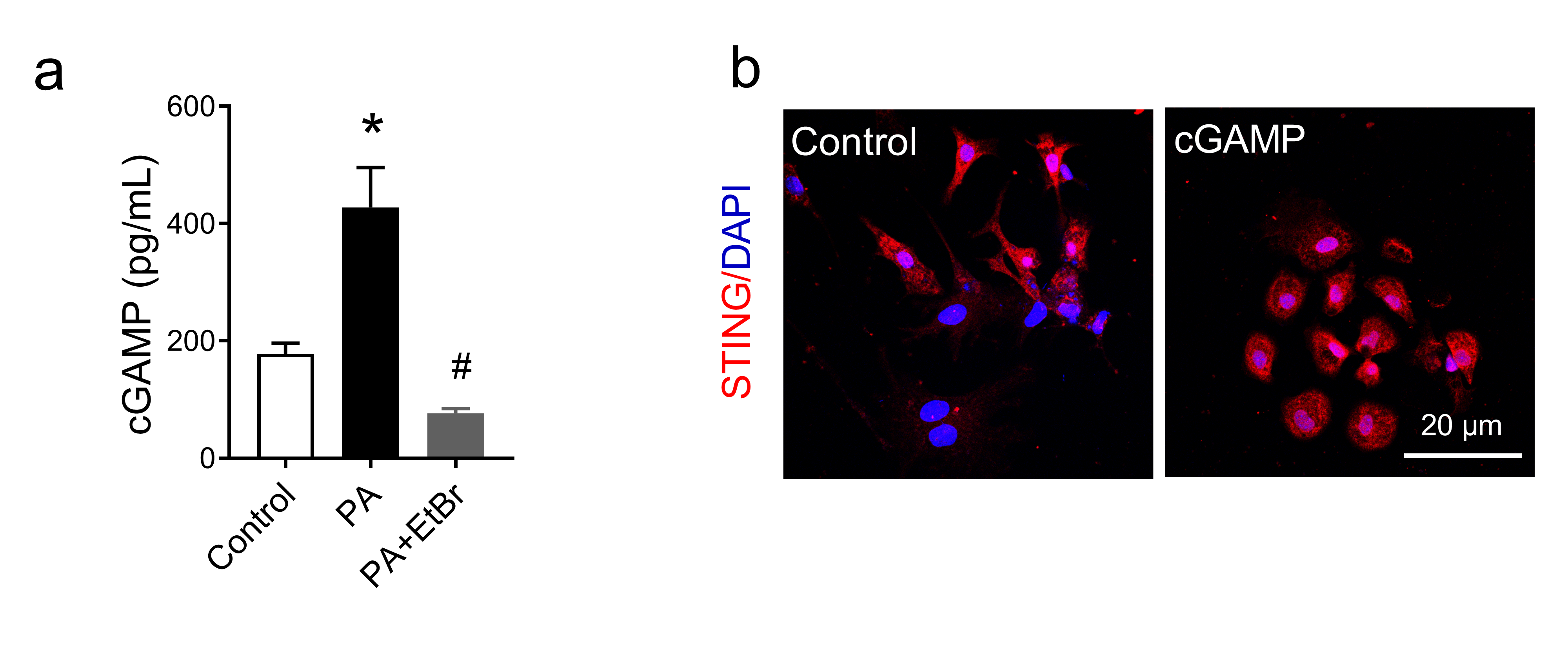

Supplement: Supplementary file 4 — figure S2 [file 41420_2022_1046_MOESM4_ESM.png]

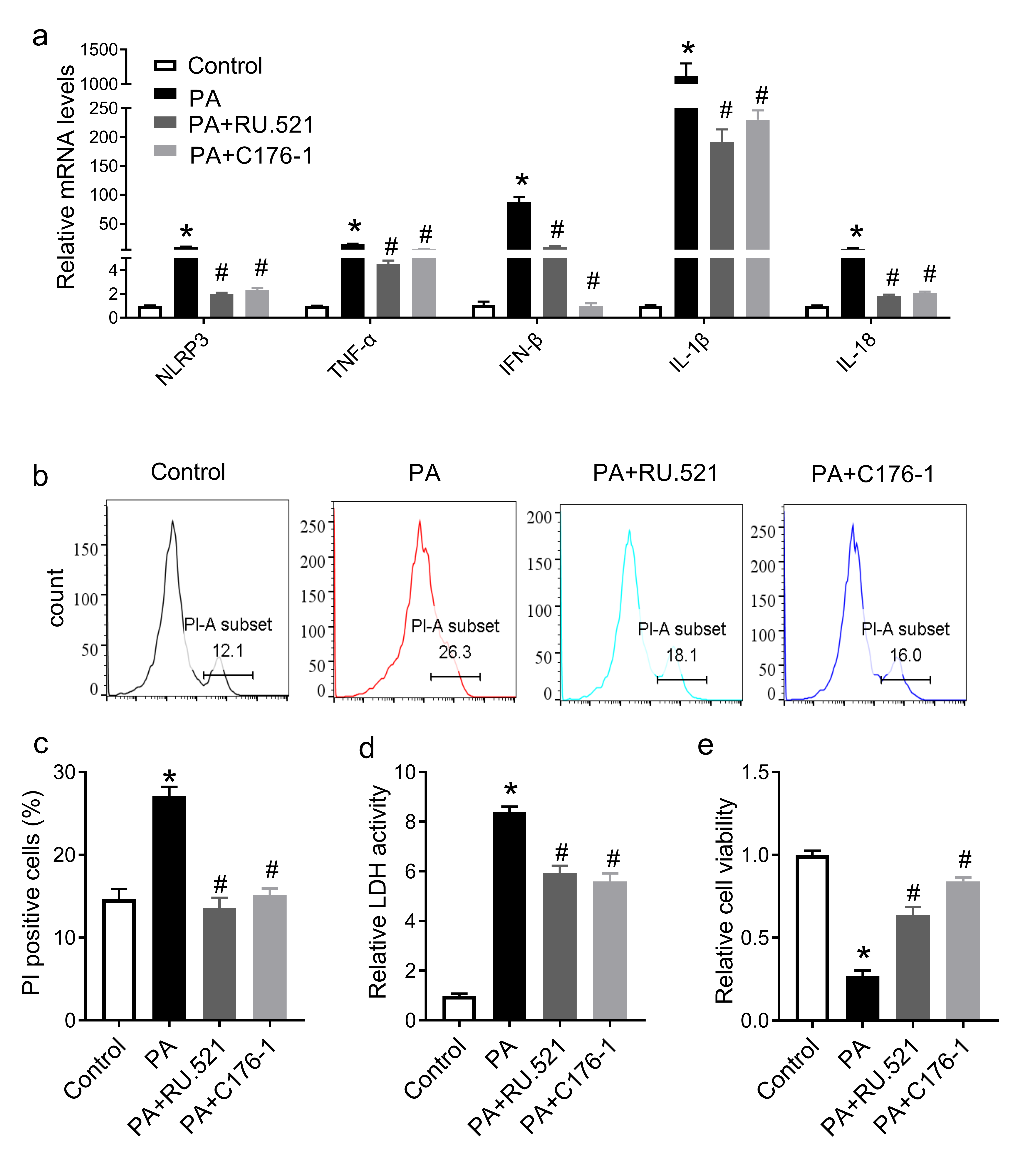

Supplement: Supplementary file 5 — figure S3 [file 41420_2022_1046_MOESM5_ESM.png]
